# Morphology Control in PDVT-10/DTCP Hybrid Films via Meniscus-Guided Cooperative Crystallization for High-Performance OFETs

**DOI:** 10.1021/acsami.5c23619

**Published:** 2026-02-04

**Authors:** Xiao-Yuan Lin, Dhananjay S. Nipate, Shih-Kang Chen, Mai Harada, U-Ser Jeng, Michal Kohout, Hong-Cheu Lin, Yasutaka Kitagawa, Tomoyuki Akutagawa, Wen-Ya Lee, Hsiu-Hui Chen

**Affiliations:** † Department of Molecular Science and Engineering, 34877 National Taipei University of Technology, Taipei 106, Taiwan; ‡ Department of Materials Engineering Science, Graduate School of Engineering Science, 320550 Osaka University, Osaka 560-8531, Japan; § 57815 National Synchrotron Radiation Research Center, Hsinchu City 300, Taiwan; ∥ Department of Organic Chemistry, University of Chemistry and Technology Prague, Prague 16628, Czech Republic; ⊥ Department of Materials Science and Engineering, National Yang Ming Chiao Tung University, Hsinchu 300093, Taiwan; # Center for Emergent Functional Matter Science, National Yang Ming Chiao Tung University, Hsinchu 300093, Taiwan; ¶ Graduate School of Engineering, 74027 Tohoku University, Sendai 980-8579, Japan; ∇ Institute of Multidisciplinary Research for Advanced Materials (IMRAM), Tohoku University, 2-1-1 Katahira, Aoba-ku, Sendai 980-8577, Japan; ○ Department of Chemical Engineering and Biotechnology, National Taipei University of Technology, Taipei 106, Taiwan

**Keywords:** meniscus-guided coating, DTCP, PDVT-10, organic field-effect transistors, photochromic dopants cooperative crystallization, molecular alignment

## Abstract

The meniscus-guided coating (MGC) method was used to prepare well-aligned films of hybrid systems composed of the conjugated polymer poly­{3,6-dithiophen-2-yl-2,5-di­(2-decyltetra-decyl)-pyrrolo­[3,4-*c*]­pyrrole-1,4-dione-*alt*-thienylenevinylene-2,5-yl} (**PDVT-10)** and a photoresponsive small molecule dopant, dithienylperfluorocyclopentene (**DTCP**), at various concentrations in their open-ring form (**DTCP-o**) or closed-ring (**DTCP-c**) form. The structures of the coated films were characterized with polarized optical microscopy (POM), grazing-incidence X-ray diffraction (GIXRD), and atomic force microscopy (AFM). The **DTCP** can undergo reversible isomerization between a more twisted open-ring form and a more conjugated closed-ring form under UV and visible light, respectively. Both **DTCP** isomers were found to function as morphology-modulating additives that facilitate cooperative crystallization, an effect attributed to enhanced solution-phase molecular association, which impacts the packing of the polymer film. Organic field-effect transistors (OFETs) were fabricated from these films. The **DTCP-c** doping progressively enhanced charge transport, reaching the highest mobility of 2.44 cm^2^ V^−1^ s^−1^ at 10 wt %. Notably, 3 wt % **DTCP-o**, typically considered insulating molecule, increased **PDVT-10** mobility from 2.12 to 3.23 cm^2^ V^−1^ s^−1^. This improvement is suggested to arise from the combined effects of precise molecular alignment by the MGC method and a favorable HOMO–HOMO energy level alignment predicted by DFT, enabling cooperative charge transfer despite the nominally insulating nature of the open-ring form. The photoswitchable **DTCP** provides a unique opportunity to optically modulate frontier molecular orbital energy levels, thereby opening up an avenue for designing electronic devices such as photocontrollable OFETs.

## Graphical abstract

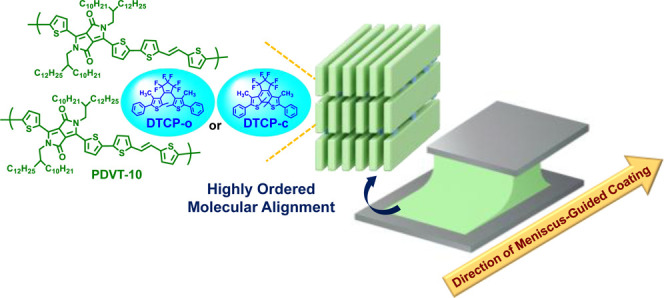

## Introduction

Organic field-effect transistors (OFETs) are promising platforms for electronics due to their flexibility, lightweight, transparency, and low fabrication cost.^1–10^ Besides optimizing charge transport through new organic semiconductors and advanced processing methods, efforts have increasingly been focused on integrating additional functionalities into OFET architectures, enabling applications in sensing, optical switching, memory, and light-emitting devices. Incorporating photoswitchable molecules into the active layer or at the semiconductor–dielectric interface offers an effective route to impart light responsiveness.^11^ These molecules, which serve as essential building blocks of molecular electronics, should be capable of significantly modulating the energy levels of the highest occupied molecular orbital (HOMO) and lowest unoccupied molecular orbital (LUMO) upon photoirradiation. Among various candidates, dithienylperfluorocyclopentene (**DTCP**) is particularly promising because its reversible photoisomerization induces pronounced changes in the electronic structure while causing only minor alterations to the molecular geometry, making it well-suited for light-tunable OFET architectures.^12–15^ These molecules switch between a nonconjugated open-ring form and a conjugated closed-ring form, giving different electronic structures, dipole moments, and energy levels in a reversible manner. Such structural transformations have been effectively utilized to modulate charge transport in OFETs. In a notable example, the research group led by Prof. Wakayama demonstrated that **DTCP** can function directly as a photoresponsive semiconducting channel material.^16,17^ Their studies showed that the closed-ring isomer of **DTCP** supports ambipolar charge transport and that alternating UV and VIS light irradiation can control both hole and electron currents. The device performance, including on/off switching ratios and carrier polarity, was determined by the energy alignment between the molecular orbitals of the closed-ring **DTCP** and the work function of the electrodes. These findings established a strong foundation for the use of **DTCP**-based molecules in optoelectronic transistors. To integrate **DTCPs** into OFETs, two major design strategies have been explored. One approach involves doping **DTCPs** into high-mobility organic semiconductors such as poly­(3-hexylthiophene) (**P3HT**), 2,7-dialkylbenzothieno­[3,2*-b*]­benzothiophene (**BTBT**), and poly­{3,6-dith-iophen-2-yl-2,5-di­(2-decyltetradecyl)-pyrrolo­[3,4*-c*]­pyrrole-1,4-dione-*alt*-thienylenevinylene-2,5-yl} (**PDVT-10**) polymers.^18–21^ In these hybrid systems, the host polymer primarily governs charge transport, while the **DTCP** molecules serve as photoresponsive dopants. However, the open-ring form of **DTCP** tends to interrupt polymer crystallinity and decrease charge mobility, which is typically limited to the range of 10^−5^ to 10^−3^ cm^2^ V^−1^ s^−1^.^22–25^ An alternative strategy employs **DTCP** molecules themselves as the active layer in the transistor channel, either as vacuum-deposited thin films or as self-assembled monolayers at the surface of gate dielectrics. Although such systems enable direct control of charge injection and transport via photoisomerization, they often suffer from poor film uniformity, weak molecular ordering, and limited operational stability. In both the doped and neat-**DTCP** configurations, the fundamental charge transport characteristics are still largely governed by the host semiconductor or by morphological constraints, and the photochromic component can become performance-limiting when incorporated at high concentrations. Recent advances in optically switchable OFETs have explored various integration architectures. One strategy involves interfacial engineering, such as functionalizing source/drain electrodes with photochromic self-assembled monolayers (SAMs) to modulate charge injection.^26^ Another approach utilizes diarylethene derivatives directly as the active channel layer to achieve ambipolar transport.^27^ However, blending photochromic dopants into semiconducting matrices remains the most versatile method. Significant progress has been made in hybrid systems, including *n*-type small-molecule/diarylethene blends^11,28^ and ambipolar polymer/diarylethene composites,^20^ where selective phototuning of charge carriers is achieved. Nevertheless, realizing high mobility in such blends is often hindered by the trade-off between crystallinity and photoswitching capability, as morphological disorder or phase separation can disrupt charge transport pathways.^13^ Therefore, developing processing methods that simultaneously promote molecular alignment and preserve dopant dispersion—such as the cooperative crystallization strategy proposed here—is critical for advancing high-performance multifunctional organic electronics.

To overcome these limitations, a **DTCP**–polymer hybrid system that combines optoelectronic functionality with precise control over molecular orientation and film morphology is desirable. For this purpose, **PDVT-10** is selected as the host semiconductor because of its favorable charge transport characteristics, good film-forming ability, and high compatibility with π-conjugated molecular additives. These features make **PDVT-10** an excellent platform for investigating morphology-dependent charge transport and photoresponsiveness in OFET applications.^21^ More recently, meniscus-guided coating (MGC) and related solution-shearing techniques have emerged as powerful tools to couple shear, capillary, and Marangoni flows near the moving contact line, thereby controlling nucleation, crystallization, and chain alignment in conjugated polymer thin films.^29–32^ This body of work suggests that thin-film morphology and anisotropic charge transport can be tuned not only through molecular design but also through careful control of flow and evaporation conditions during coating. In the present study, this solution-shearing strategy is applied to fabricate hybrid thin films composed of **PDVT-10** and **DTCP** derivatives to explore the effect of photochromic **DTCP** moiety on the morphology and charge transport in the polymer-based OFETs.

## Experimental Section

### Materials and Methods

All chemicals were used as received without further purification. The 1,2-bis­(2,4-dimethyl-5-phenyl-3-thienyl)-3,3,4,4,5,5-hexafluoro-1-cyclopentene (**DTCP-o**) was obtained from Tokyo Chemical Industry Co., Ltd. (TCI), 99.0%. Reversible isomerization between the open-ring and closed-ring forms of **DTCP** was achieved by sequential exposure to UV light at 313 nm and visible light at 580 nm, enabling dynamic optical switching under ambient conditions. The *n*-octadecyltrimethoxysilane (OTMS) (Gelest, 92.0%), **PDVT-10** (Luminescence Technology Corp, M.W. > 30,000), hexane (ECHO, 95%), chloroform (Macron, 99.8%), and anhydrous toluene (Aldrich, 99.8%) were acquired from respective sources. Acetone (99.5%), isopropanol (99.5%), and ammonia solution (NH_4_OH_(aq)_) (28–30%) were purchased from Mallinckrodt. The MGC polymer films were inspected using a Nikon Eclipse LV100N polarizing optical microscope (POM). Surface morphology was examined with a Park XE-100 atomic force microscope (AFM) operated in tapping mode. The elemental distribution of the films was characterized by energy-dispersive X-ray spectroscopy (EDX) mapping. These measurements were conducted using a Hitachi TM4000Plus scanning electron microscope (Tokyo, Japan) at an accelerating potential of 15 kV. Ultraviolet–visible (UV–vis) absorption spectra were recorded using a Hitachi UH5300 spectrophotometer (Tokyo, Japan) over a wavelength range of 200–1000 nm. For solid-state characterization, polarized UV–vis spectra of the films were obtained to investigate optical anisotropy. For solution-phase analysis, specifically to investigate the concentration evolution during the solvent evaporation process, absorption spectra of **DTCP-o**, **PDVT-10**, and their hybrids dissolved in chloroform were measured. A concentration series ranging from 1 × 10^−6^ M to 1.32 × 10^−4^ M was prepared to establish the correlation between absorbance and concentration. Dynamic light scattering (DLS) measurements for particle size analysis were conducted on a Brookhaven Nanobrook 90Plus PALS instrument. The polymer and hybrid solutions were prepared in chloroform at a concentration of 2.75 × 10^−7^ M. Grazing incidence X-ray diffraction (GIXRD) measurements were performed at the Beamline 13A of the Taiwan Light Source in the National Synchrotron Radiation Research Center (NSRRC, Hsinchu, Taiwan), using a grazing incident angle of 0.12°. The sample-to-detector distance was approximately 27.7 cm, calibrated using Ag behenate as a standard. To minimize geometric errors in peak positions, a precise height alignment was performed for each individual sample, positioning the film surface at the center of the goniometer rotation prior to exposure. The thickness of the **PDVT-10** and **PDVT-10/DTCP** hybrid films was measured to be approximately 110 ± 7 nm (summarized in Table S1 in the Supporting Information). Although the 2D-GIXRD patterns are presented in detector coordinates to preserve signal clarity, all quantitative data (*d*-spacing, fwhm, and coherence length) were extracted from 1D profiles after rigorous conversion from pixel coordinates to the scattering vector q. The 1D line profiles were obtained by performing azimuthal integration over a 60° wedge centered at the meridional (out-of-plane, χ ≈ 90° ± 30°) and equatorial (in-plane, χ ≈ 180° ± 30°) directions.

Density functional theory (DFT) calculations were performed using the Gaussian 09 (rev. D01) package at the B3LYP/6-31G level in the gas phase. Molecular geometries of **PDVT-10**, **DTCP-o,** and **DTCP-c** were optimized using initial structures derived from AM1 (for **PDVT-10**) and single-crystal data (#155979 and #155980, for **DTCP-o** and **DTCP-c**, respectively). All optimized structures were confirmed to have no imaginary frequencies, indicating that they are true minima on the potential energy surface.^33^ The results are summarized in Table S2 in the Supporting Information.

### MGC Process of PDVT-10 and PDVT-10/DTCP Films

The **DTCP** and **PDVT-10** (structures shown in Figure 1a,b) were dissolved in chloroform at concentrations of 5 and 10 mg/mL, respectively. The **DTCP** solution was blended with **PDVT-10** solution at weight percentages of 1, 3, 5, 10 wt % to give compositions denoted as **PDVT-10/1 wt % DTCP**, **PDVT-10/3 wt % DTCP**, **PDVT-10/5 wt % DTCP**, and **PDVT-10/10 wt % DTCP**. These resulting solutions were homogenized by stirring followed by sonication. A 7 μL aliquot of **PDVT-10** solution was deposited between two precisely aligned silicon substrates, where the upper substrate, functioning as a movable blade, was tilted at a fixed angle of 7° relative to the stationary lower substrate placed on a hot plate maintained at 40 °C, as shown in Figure 1c. To establish optimal coating conditions for molecular alignment, systematic tests were first conducted using **PDVT-10** under various processing parameters. Specifically, the blade movement speed was adjusted across at 200, 300, 400, 500, and 600 μm/s; the intersubstrate gap was varied among 0.05, 0.07, 0.09, and 0.11 mm; and the solution concentration was tested at 3, 5, 8, and 10 mg/mL. These tests enabled the identification of the most favorable alignment conditions, which were then applied to fabricate hybrid films composed of **PDVT-10** and different weight percentages of **DTCP-o** or **DTCP-c** for subsequent morphological and electrical characterization.

**Figure 1:**
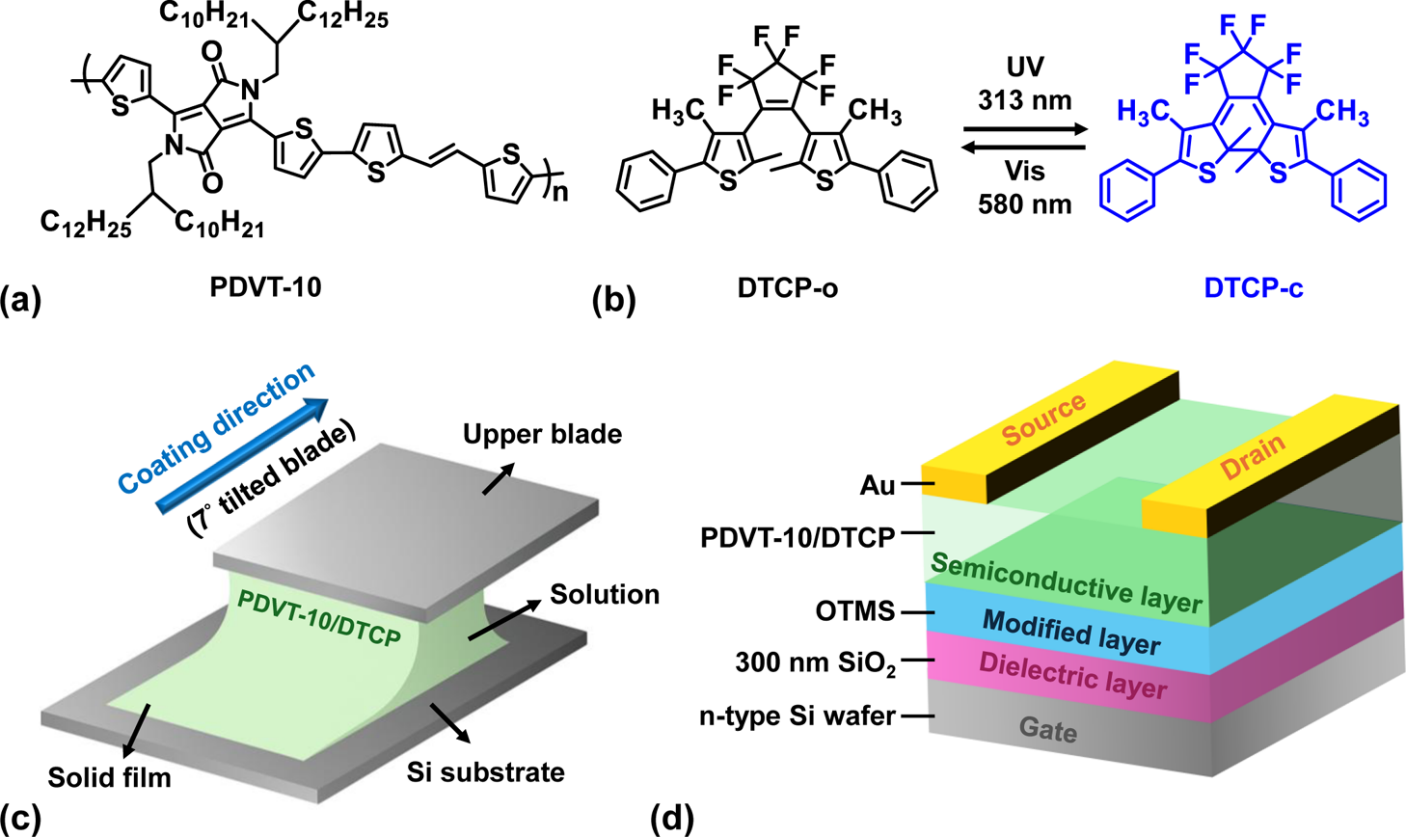
Molecular structures of (a) **PDVT-10** and (b) the open- and closed-form isomers of **DTCP**; (c) schematic description of the MGC process; (d) illustration of the bottom-gate/top-contact MGC OFET devices.

### Fabrication of OFETs

The bottom-gate top-contact OFET devices were fabricated. The n-type silicon wafers were used as the gate electrode with a 300 nm-thick thermally grown SiO_2_ layer as the dielectric. The wafers were modified with a monolayer of OTMS according to a known procedure, by treating with plasma for 5 min, followed by surface modification via spin-coating a 3 mM OTMS solution (in anhydrous toluene) at 3000 rpm.^32,33^ The OTMS-treated wafer was then exposed to an ammonia vapor overnight at room temperature to promote the growth of a crystalline OTMS monolayer. After that, the wafers were rinsed with toluene and then cleaned with acetone using sonication for 10 min, followed by sequential washing with toluene, acetone, and isopropanol. Residual solvents were removed by blowing nitrogen gas. The semiconductive layer was then deposited on top via the meniscus-assisted solution coating process. Source and drain electrodes (80 nm-thick-Au) were deposited by the thermal evaporation method through a shadow mask (Figure 1d).

## Results and Discussion

### Molecular Alignment of PDVT-10 and DTCP-Blended Films Prepared via MGC Process

To establish suitable processing conditions for film alignment, the MGC method was first optimized using only **PDVT-10** as the model system. Coating was performed on wafers with CHCl_3_ as the solvent and the substrate held at 40 °C. Systematic variation of parameters identified a coating speed of 500 μm/s, a blade gap of 0.09 mm, and a solution concentration of 5 mg/mL as the conditions that yielded the most uniform molecular alignment, as evidenced by OM and POM (Figures S1–S3, Supporting Information in ESI‡). The optimized coating speed range between 200–600 μm/s was identified as the transition regime where meniscus stability is maximized. At slower speeds below 100 μm/s corresponding to the evaporation regime, stick–slip motion resulted in discontinuous and overly thick films with reduced alignment. Conversely, at faster speeds exceeding 1000 μm/s within the Landau–Levich regime, the wet liquid film dried isotropically postcoating, failing to preserve the shear-induced chain orientation.^29^ The same conditions were then applied to **PDVT-10** films blended with various amounts of **DTCP-o** and **DTCP-c**, with the results shown in Figures 2 and S4 and S5. Figure 2 shows the best performing examples: pristine **PDVT-10**, **PDVT-10** with 3 wt % **DTCP-o**, and **PDVT-10** with 10 wt % **DTCP-c**. In OM, the bare wafer appears green, whereas the coated regions display yellow-orange stripe patterns. In POM, the inset labels the analyzer (*A*), the polarizer (*P*), and the red line corresponding to the λ = 530 nm retardation plate. Upon rotating the stage by ± 45°, alternating red and dark green birefringent stripes become evident against a purple background that originates from the wafer viewed through the retardation plate. Taken together, these observations indicate highly ordered alignment of the films on the wafer surface.

**Figure 2:**
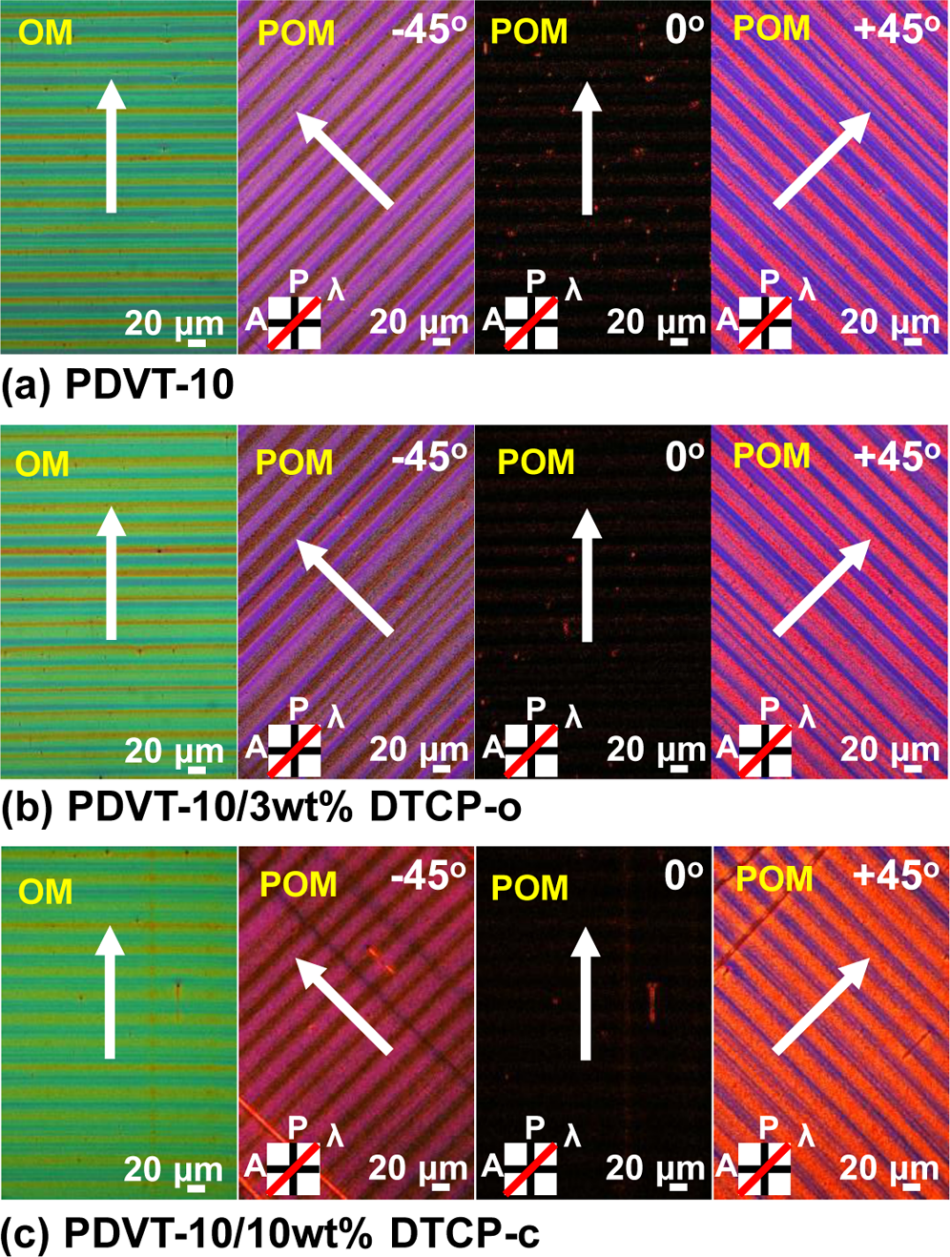
OM and POM images of (a) neat **PDVT-10** (b) **PDVT-10/3 wt % DTCP-o**, and (c) **PDVT-10/10 wt % DTCP-c** thin films, recorded under different polarization angles (0°, ± 45°) using a λ = 530 nm retardation plate. The white arrow indicates the coating direction.

### Molecular Orientation and Crystallographic Alignment in PDVT-10/DTCP Hybrid Films

The molecular packing and crystalline orientation of the coated **PDVT-10** and **PDVT-10/DTCP** thin films were examined by 2D-GIXRD. In this setup, the incident X-ray beam impinges on the sample surface either along or perpendicular to the coating direction, and the scattered intensity is collected in the out-of-plane (*q*_*z*_) and in-plane (*q*_*x,y*_) directions. Figure 3 schematically illustrates the measurement geometry and lamellar stacking relative to the coating direction; yellow arrows indicate the coating direction. The azimuthal sectors used for integration are defined as perpendicular (⊥) and parallel (∥) with respect to the coating direction. Representative patterns for each sector show lamellar reflections at d (100), d (200), and d (300), and additional data sets are provided in Figures S6–S9. In addition to the characteristic **PDVT-10** lamellar diffraction at d (100), higher-order reflections at d (200) and d (300) were observed in all blended films, indicating enhanced molecular ordering. Diffraction features were converted to real-space distances using Bragg’s law,^34^ and the results—analyzed separately for the ⊥ and ∥ sectors—are summarized in Tables 1 and 2. For both **DTCP-o** and **DTCP-c** blends over a range of concentrations, the lamellar spacing d (100) increases beyond the pristine **PDVT-10** values of 21.53 Å (⊥) and 21.61 Å (∥), consistent with expansion of the layered structure upon blending. Notably, at the low concentration of 1 wt %, a deviation is observed where lattice expansion occurs primarily in the perpendicular direction, while the parallel spacing remains constrained. This is attributed to the dominant shear stress along the coating direction, which suppresses lamellar swelling until a critical dopant threshold of approximately 3 wt % is reached. This behavior aligns with previous reports on shear-induced anisotropic lattice strain.^35,36^ Concurrently, the fwhm decreases from about 0.5 to about 0.4, indicating improved structural order despite the larger spacing. In contrast, the π–π stacking distance d (010) remains nearly constant at ∼3.5 Å for all compositions and in both sectors, showing that incorporation of **DTCP** does not interrupt the intrinsic stacking of **PDVT-10**; in some cases, d (010) even slightly decreases. Notably, although **DTCP-o** has a nonplanar geometry and a relatively larger molecular volume, d(010) values of the polymer chains remain within 3.52–3.56 Å in both orientations. Among all compositions, the **3 wt % DTCP-o** blend exhibits the most pronounced ordering in the ⊥ sector. Using the Scherrer equation (see in ESI‡),^37^ the coherence lengths *L* were extracted; for the 3 wt % **DTCP-o** blend, *L*⊥ ≈ 134.87 Å exceeds *L*∥ ≈ 125.01 Å, and both are greater than the corresponding value for pristine **PDVT-10** (for example, *L* ≈ 39.91 Å for the same reflection). As shown in Figure 4a, the integrated diffraction intensity of **PDVT-10** blended with 1–3 wt % **DTCP-o** for the ⊥ sector is consistently higher than that for the ∥ sector across all blending ratios, suggesting a preferential edge-on orientation. This is further supported by the orthogonal distribution of diffraction vectors, where the d (h00) lamellar peaks dominate the out-of-plane profiles while the (010) π–π stacking peak is predominantly observed in the in-plane profiles. Such an edge-on texture creates efficient hole-transport channels parallel to the substrate, which is essential for high-performance OFETs. Figure 4b presents a plausible molecular packing model inferred from the foregoing GIXRD results. In this model, **PDVT-10** adopts a predominant edge-on lamellar packing, while **DTCP-o** resides in the alkyl side-chain regions, increasing d (100) without perturbing the π–π stacking.

**Figure 3:**
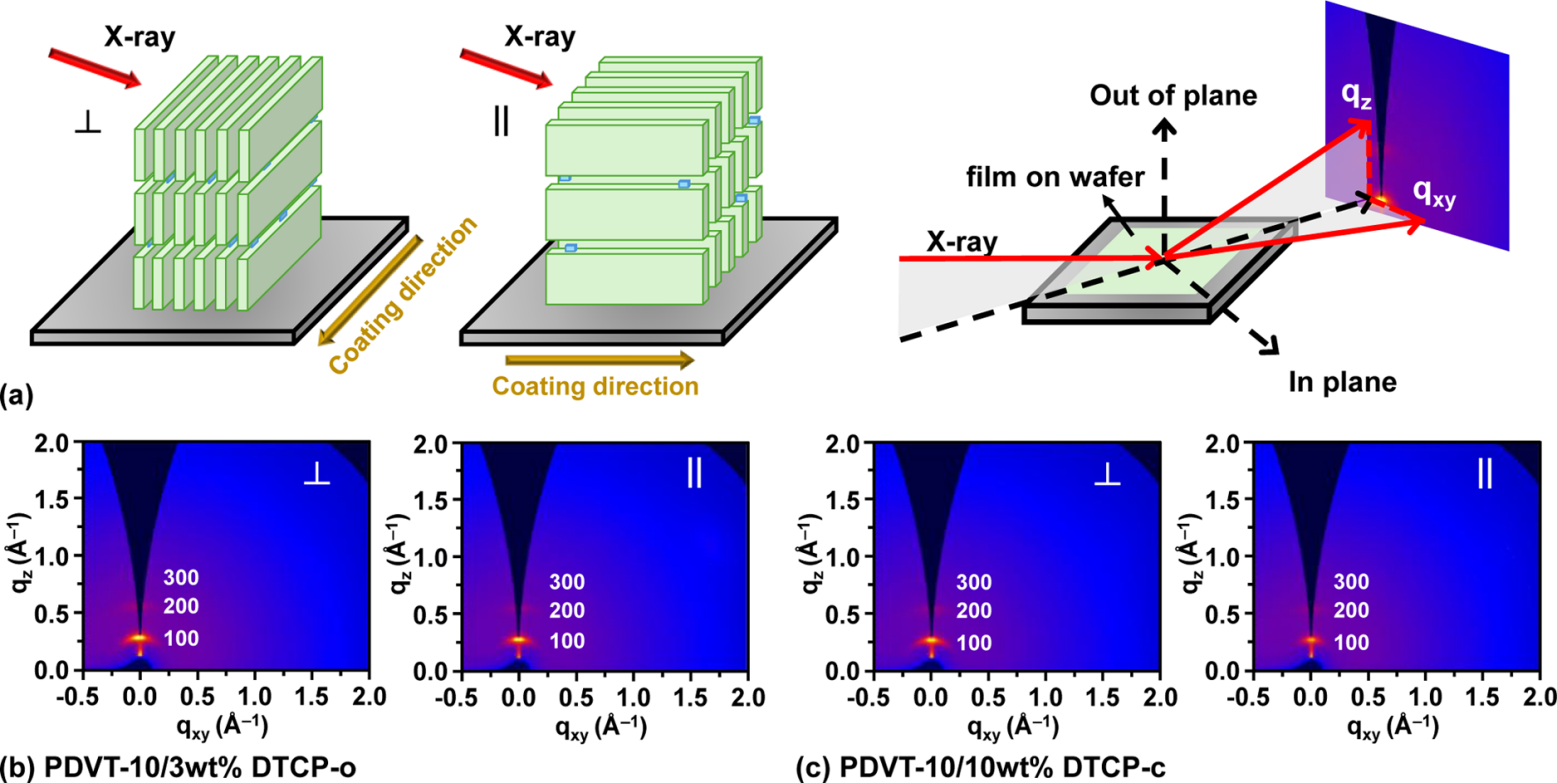
Illustration of (a) the perpendicular (⊥) and parallel (||) orientations of the incident X-ray beam with respect to the coating direction at an incidence angle of 0.12°. (b) Schematic 2D GIXRD pattern of **PDVT-10/3 wt % DTCP-o**. (c) **PDVT-10/10 wt % DTCP-c**.

**Table 1:** Crystallographic Information Extracted by GIXRD Measurements of the Coated **PDVT-10/DTCP-o** Thin Films Deposited on Wafers

coating direction	samples	d (100) lamellar spacing (Å)	d (100) peak fwhm (1/Å)	*L*_c, lamellar_ (Å)	π–π stacking distance (Å)	d (010) peak fwhm (1/Å)	*L*_c_, _π–π_ (Å)
⊥	**PDVT-10**	21.53	0.05	121.99	3.57	0.15	37.32
	**PDVT-10/1 wt % DTCP-o**	21.81	0.04	134.64	3.56	0.15	38.82
	**PDVT-10/3** **wt % DTCP-o**	21.80	0.04	134.87	3.57	0.14	39.91
	**PDVT-10/5 wt % DTCP-o**	22.13	0.04	128.96	3.57	0.16	34.56
	**PDVT-10/10 wt % DTCP-o**	22.49	0.04	129.65	3.56	0.17	32.95
||	**PDVT-10**	21.61	0.05	124.07	3.55	0.17	33.89
	**PDVT-10/1 wt % DTCP-o**	21.58	0.04	129.86	3.54	0.15	36.53
	**PDVT-10/3** **wt % DTCP-o**	21.74	0.04	125.01	3.52	0.15	37.13
	**PDVT-10/5** **wt % DTCP-o**	22.06	0.04	125.88	3.52	0.15	36.87
	**PDVT-10/10 wt % DTCP-o**	22.55	0.04	125.88	3.55	0.26	21.82

**Table 2:** Crystallographic Information Extracted by GIXRD Measurements of the **PDVT-10/DTCP-c** Thin Films Deposited on Wafers

coating direction	samples	d (100) lamellar spacing (Å)	d (100) peak fwhm (1/Å)	*L*_c, lamellar_ (Å)	π–π stacking distance (Å)	d (010) peak fwhm (1/Å)	*L*_c_, _π–π_ (Å)
⊥	**PDVT-10**	21.53	0.05	121.99	3.57	0.15	37.32
	**PDVT-10/1** **wt % DTCP-c**	21.83	0.04	132.22	3.59	0.17	33.45
	**PDVT-10/3** **wt % DTCP-c**	21.93	0.04	134.00	3.59	0.15	37.24
	**PDVT-10/5** **wt % DTCP-c**	22.26	0.04	129.32	3.57	0.16	34.32
	**PDVT-10/10** **wt % DTCP-c**	22.30	0.04	143.74	3.55	0.17	37.62
||	**PDVT-10**	21.61	0.05	124.07	3.55	0.17	33.89
	**PDVT-10/1** **wt % DTCP-c**	21.71	0.04	129.65	3.57	0.12	46.15
	**PDVT-10/3** **wt % DTCP-c**	21.86	0.05	121.19	3.56	0.13	43.53
	**PDVT-10/5 wt % DTCP-c**	22.08	0.05	114.45	3.55	0.13	43.75
	**PDVT-10/10** **wt % DTCP-c**	22.32	0.04	145.84	3.55	0.11	49.43

**Figure 4:**
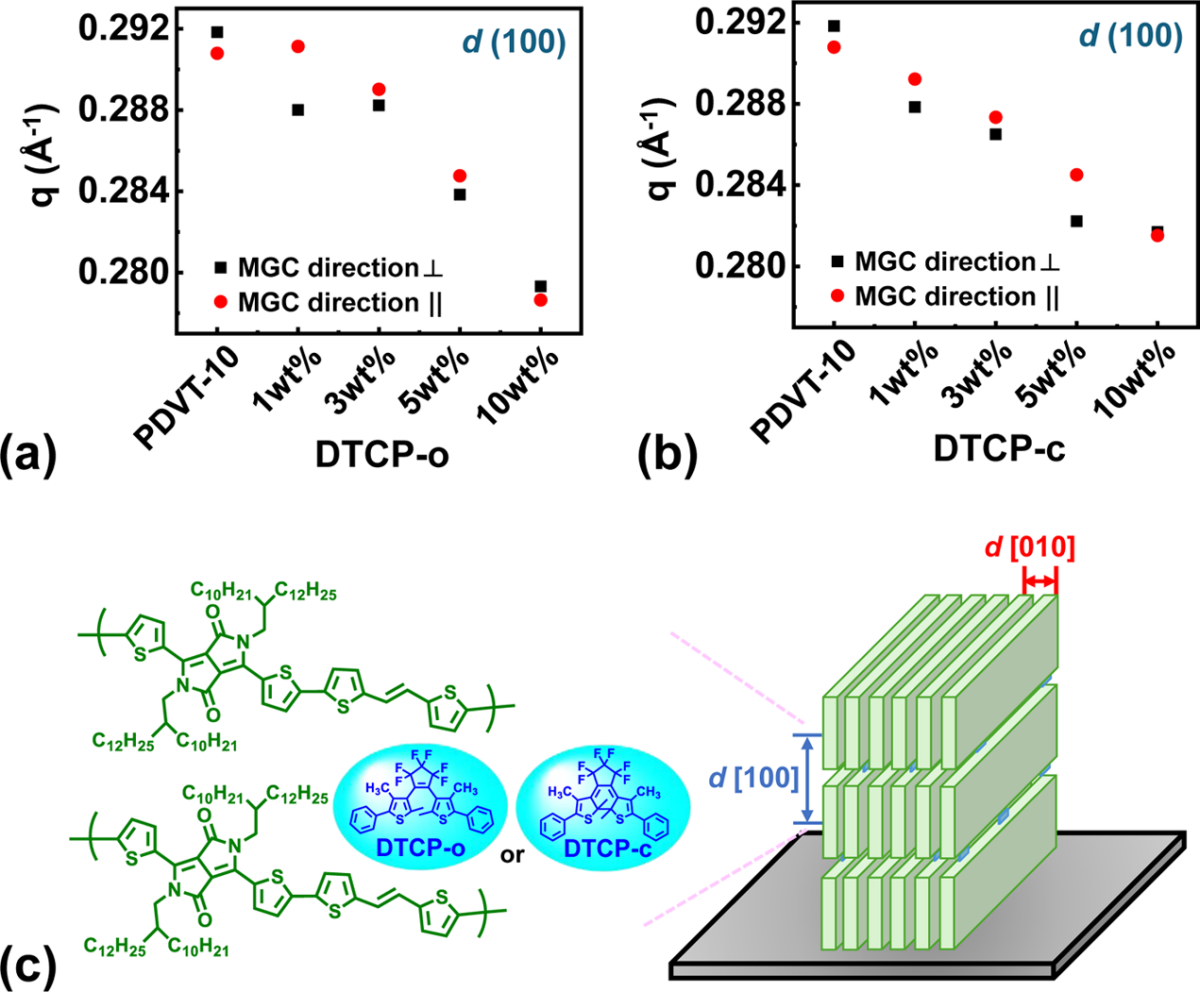
Variation of the lamellar d (100) scattering vector (q) as a function of dopant concentration for (a) **PDVT-10/DTCP-o** and (b) **PDVT-10/DTCP-c** hybrid films. The black squares and red circles represent measurements extracted from GIXRD profiles along the directions perpendicular (⊥) and parallel (||) to the MGC direction, respectively. (c) Schematic of the MGC crystal polymorph crystalline film on a wafer for **PDVT-10** and **PDVT-10/DTCP-o** or **PDVT-10/DTCP-c**, showing orientation to the edge-on form.

To visualize the microstructure heterogeneity beyond a single average value, the crystallite size distribution was simulated based on the L derived from the Scherrer equation, as shown in Figure S10. It is widely accepted that grain growth processes in polycrystalline materials typically follow a log-normal distribution rather than a Gaussian distribution. Since the X-ray diffraction intensity is proportional to the scattering volume, a volume-weighted log-normal probability density function was employed (eq 1)­

1 $$P\left(D\right)=\frac{1}{D\sigma \sqrt{2\pi }}\exp\left(-\frac{{\left(\ln D-\mu \right)}^{2}}{{2\sigma }^{2}}\right)$$

where *D* is the crystallite size, and μ and σ are the location and scale parameters (log-scale mean and standard deviation), respectively. The *L* is mathematically equivalent to the volume-weighted mean of the distribution. To reconstruct the distribution, the parameter μ was calculated by rearranging the moment equation for a log-normal distribution (eq 2)­

2 $$\mu =\ln\left(L\right)—0.5\sigma^{2}$$

A constant shape parameter of σ = 0.3, representative of polycrystalline thin films reported in the literature,^38,39^ was adopted for all samples to enable consistent comparison across compositions. Importantly, the reconstructed distributions reveal a systematic shift toward larger dominant crystallite sizes, together with a narrowing of the distribution, upon **DTCP** incorporation. These features indicate enhanced structural uniformity and promoted grain growth within the **PDVT-10** matrix. Such behavior is consistent with nucleation-assisted or aggregation-mediated crystallization pathways. To further investigate the origin of this enhanced crystallization, we probed the solution-phase behavior using DLS. As shown in Figure S10, the neat **PDVT-10** solution exhibits a baseline hydrodynamic diameter (*D*_h_) of 3.23 × 10^4^ nm. Upon the incorporation of **DTCP**, a pronounced increase in aggregate size is observed: the *D*_h_ increases to 9.95 × 10^4^ nm for the 3 wt % **DTCP-o** blend and further expands to 1.80 × 10^5^ nm for the 10 wt % **DTCP-c** blend. This systematic enlargement indicates that **DTCP** promotes the preassociation of polymer chains in the solution state. These larger, solution-phase aggregates likely serve as ordered precursors that facilitate the cooperative crystallization and domain growth during the meniscus-guided coating process, consistent with the enhanced crystallite sizes observed in GIXRD.

### Surface Morphology Characterization

AFM was employed to investigate the surface morphology and roughness of **PDVT-10** thin films blended with **DTCP**, including representative compositions with different wt % of **DTCP-o** and **DTCP-c**, as shown in Figure 5. Upon **DTCP** incorporation, both the arithmetic average roughness (*R*_a_) and the root-mean-square roughness (*R*_q_) are markedly reduced compared to pristine **PDVT-10**, indicating the formation of a smoother and more uniform surface morphology. Notably, the **PDVT-10/10 wt % DTCP-c** film exhibits the lowest *R*_a_ and *R*_q_ values, corresponding to the highest degree of surface uniformity among all samples. Because *R*_q_ is particularly sensitive to large height fluctuations, its pronounced decrease suggests effective suppression of out-of-plane height variations and mesoscale surface roughness induced by **DTCP** addition. Morphologically, **DTCP**-containing films exhibit more continuous and laterally coalesced stripe-like domains aligned along the coating direction, rather than increased vertical height aggregation. The enhanced stripe continuity and in-plane domain connectivity give rise to locally brighter contrast in AFM images while simultaneously reducing overall surface roughness. Notably, similar morphological characteristics are consistently observed across all **DTCP** concentrations investigated, with no AFM evidence of secondary phases or additive-rich aggregates.

**Figure 5:**
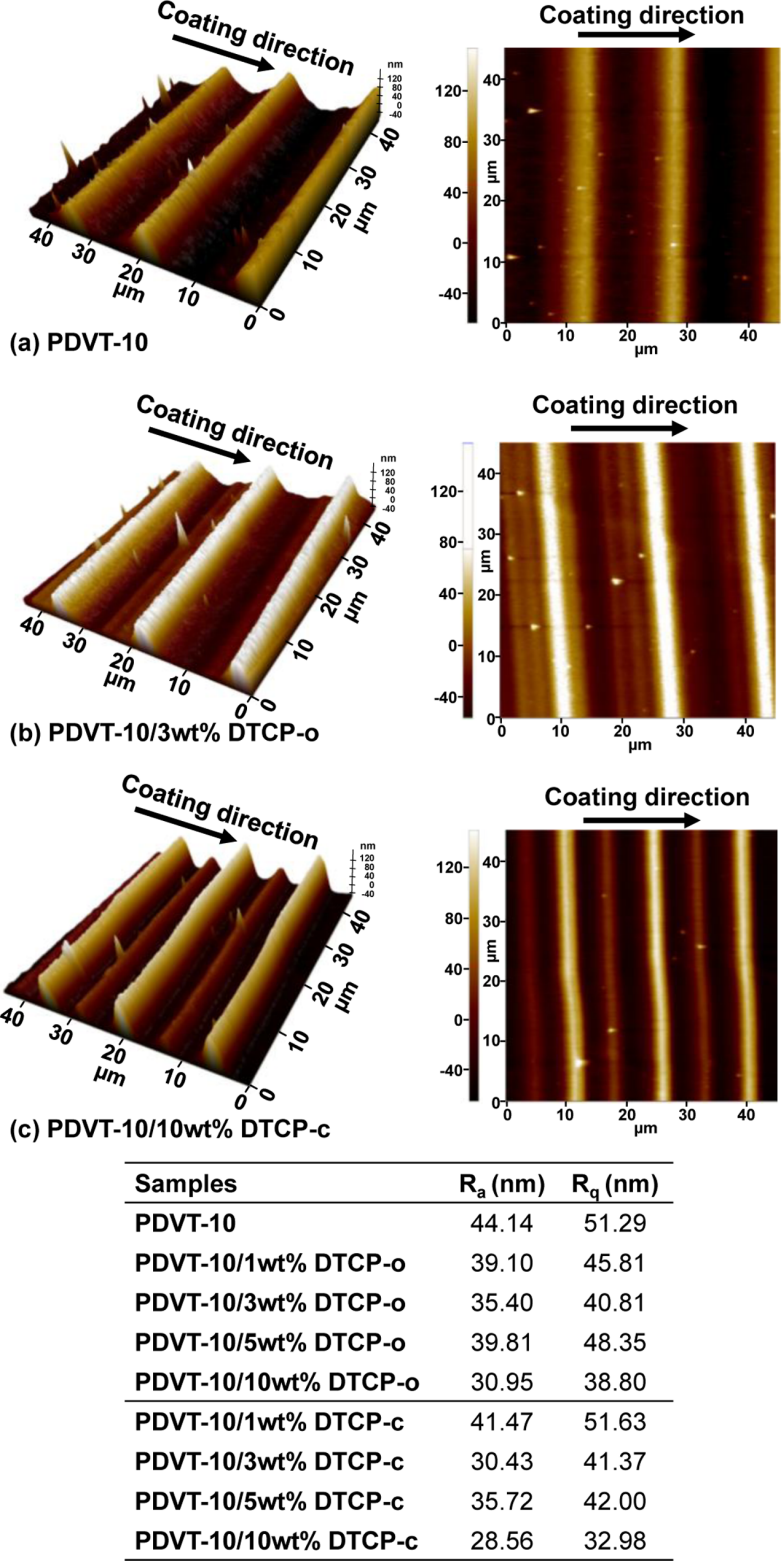
AFM micrograph of (a) **PDVT-10**, (b) **PDVT-10/3 wt % DTCP-o**, and (c) **PDVT-10/10 wt % DTCP-c** films.

To further verify whether **DTCP** exhibits local enrichment or phase separation, energy-dispersive X-ray (EDX) mapping was performed on a representative **PDVT-10/3 wt % DTCP** film, in which **DTCP**-induced morphological modulation is clearly observed while maintaining good film uniformity (in Figure 6). Here, EDX is employed as a qualitative probe to assess the spatial homogeneity of **DTCP** rather than to establish composition-dependent trends. The fluorine signal, uniquely associated with **DTCP**, is found to be uniformly distributed across the film surface, with no indication of localized **DTCP**-rich domains. This observation is fully consistent with the AFM height and phase images, confirming that the observed contrast variations originate from enhanced lateral molecular organization rather than additive precipitation. Overall, the combined AFM and EDX analyses demonstrate that **DTCP** remains homogeneously dispersed within the **PDVT-10** matrix and functions as a morphology-modulating additive that enhances in-plane ordering and surface uniformity under meniscus-guided coating conditions.

**Figure 6:**
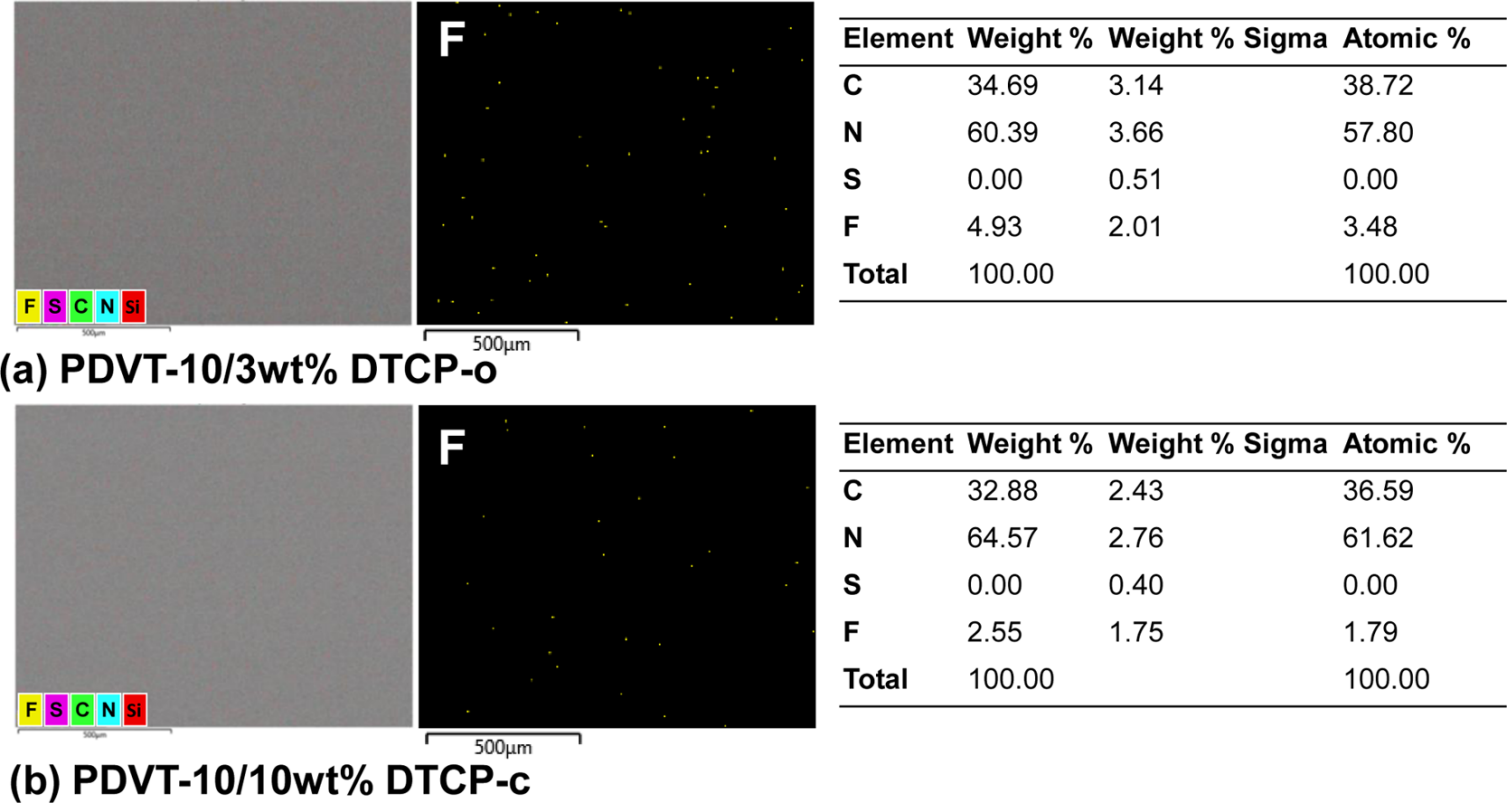
Surface morphology and the EDX elemental mapping of the (a) **PDVT-10/3 wt % DTCP-o** and (b) **PDVT-10/10 wt % DTCP-c** films showing the distribution of C, N, S, and F elements.

### Optical Anisotropy and Alignment Analysis via Polarized UV–Vis Spectroscopy

To investigate the position-dependent evolution of molecular alignment during the MGC process, we performed position-dependent polarized UV–vis absorption spectroscopy at three distinct regions along the coating direction: the Start (I), Middle (II), and End (III) zones (Figure 7a). As shown in Figures 7b–d, the absorption spectra measured with light polarization parallel (∥) and perpendicular (⊥) to the coating direction reveal a progressive enhancement in optical anisotropy from the initial contact line to the terminal region for all films. Quantitatively, the dichroic ratio (*D*) exhibits a clear lateral gradient. In the Start zone (I), the films show relatively low *D* values (1.05–1.23), indicating limited alignment. This is attributed to the transient nature of the meniscus formation at the leading edge, where unstable contact line dynamics (such as stick–slip motion) disrupt the effective shear field. As the coating progresses to the Middle (II) and End (III) zones, the meniscus transitions into a fully developed steady-state regime. In this stabilized flow environment, the constant evaporation and continuous solution supply allow the shear stress to effectively align the polymer chains. Consequently, the End zone (III) consistently exhibits the highest molecular ordering, with *D* values reaching a maximum (*D* = 3.79 for the 3 wt % hybrid film). This systematic increase in alignment (*D*_Start_ < *D*_Middle_ < *D*_End_) provides compelling evidence for the proposed cooperative crystallization mechanism. It suggests that the templating effect of **DTCP** is cumulative and relies on the establishment of a stable meniscus to fully propagate. The superior alignment observed in the **PDVT-10/3 wt % DTCP-o** film at the terminal region further confirms that, under optimized steady-state conditions, **DTCP** molecules effectively modulate the packing energetics to lock in the shear-induced orientation, minimizing the relaxation of polymer chains during solidification.

**Figure 7:**
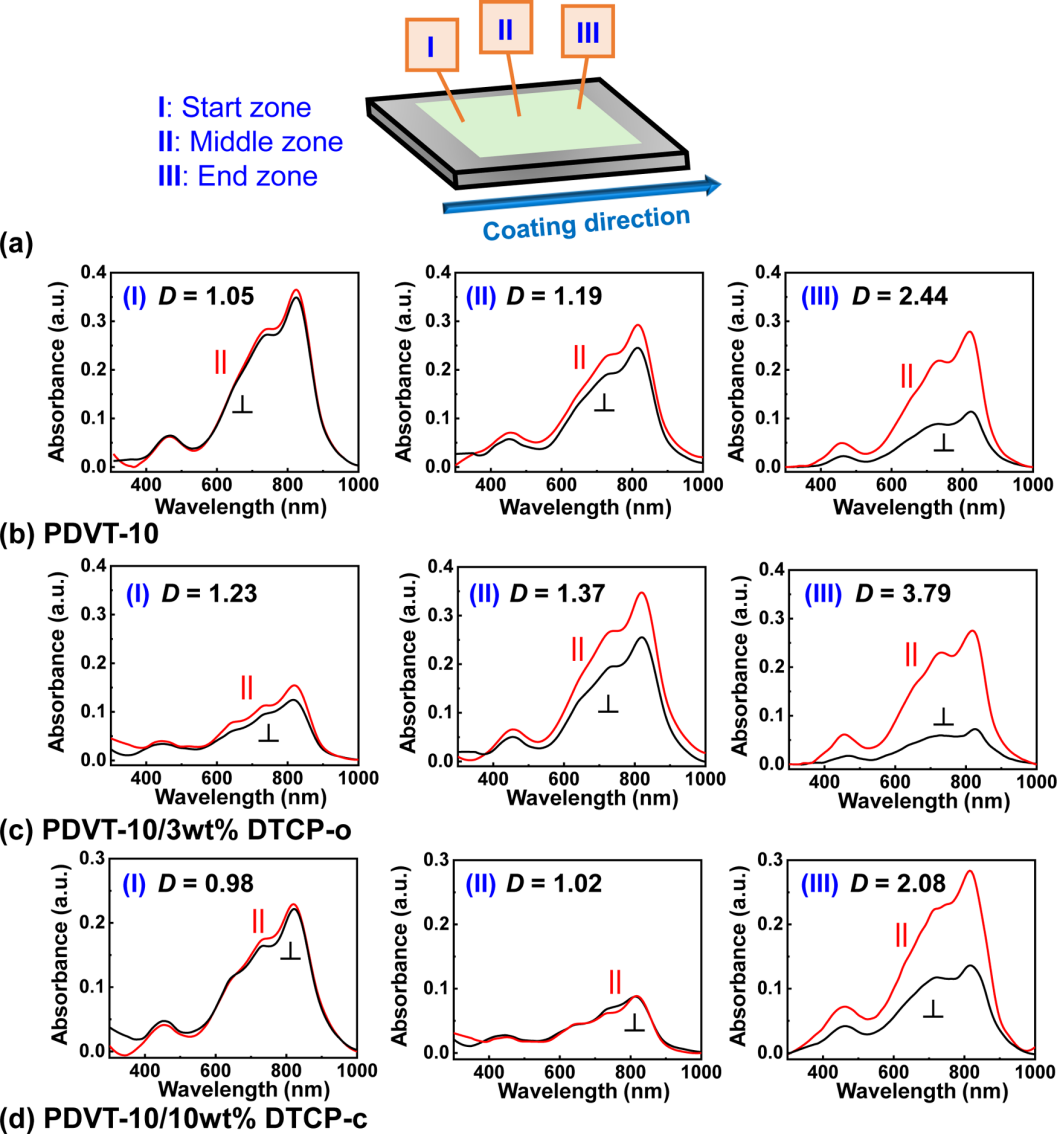
(a) Schematic illustration of the MGC process, defining the Start (I), Middle (II), and End (III) zones along the coating direction. Position-dependent polarized UV–vis absorption spectra recorded at these corresponding zones for (b) neat **PDVT-10**, (c) **PDVT-10/3 wt % DTCP-o**, and (d) **PDVT-10/10 wt % DTCP-c** films. The red and black curves correspond to absorption measured with the light polarization parallel (∥) and perpendicular (⊥) to the coating direction, respectively.

### DTCP Regulated Cooperative Crystallization during MGC

As illustrated in Figure 8, a **DTCP**-regulated, shear- and evaporation-coupled cooperative crystallization framework is proposed to rationalize the evolution of molecular ordering and mesoscale morphology in meniscus-guided coated **PDVT-10** films. This model is anchored by a coherent set of experimental observations. First, the GIWAX-derived crystallite or ordered coherence analysis reveals an increased characteristic ordering length scale upon **DTCP** addition, and this trend is independently corroborated by DLS, which shows a concomitant increase in solution-phase aggregate size. This quantitative evidence is visually reinforced by Tyndall effect experiments (Figure S15), where the **DTCP** solution exhibits negligible light scattering, consistent with a molecularly dispersed state. In contrast, the **PDVT-10** solution displays a distinct laser path, confirming the presence of preaggregated polymer precursors. Collectively, these complementary probes indicate that **DTCP** shifts the precursor state toward a more aggregated and structurally correlated regime prior to solidification. Related small-molecule blended conjugated polymer systems have been discussed in the literature and are summarized in Table 3;^11,13,20,26–28,37,40^ however, explicit definitions that connect the roles of each component with solvent choice, coating speed, and substrate temperature are still limited for such hybrid systems. Therefore, based on the present data set, a simplified working mechanism is formulated.

**Figure 8:**
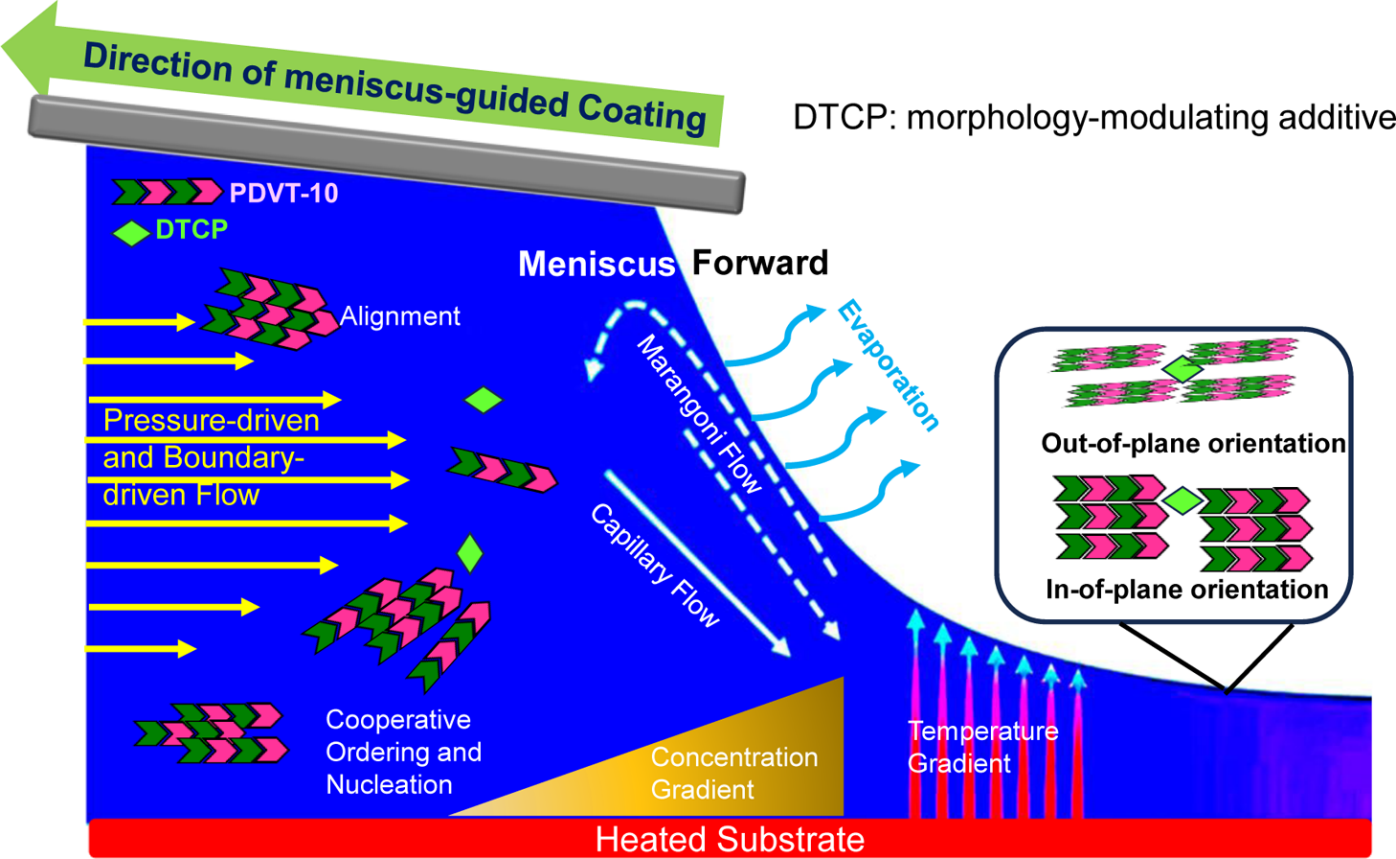
Schematic illustration of the proposed cooperative crystallization mechanism in **PDVT-10/DTCP** blend films during MGC, where coupled shear and evaporation align **PDVT-10** fibrils while **DTCP-o/DTCP-c** act as morphology-modulating additives, leading to aligned lamellae, controlled domain structure, and enhanced in-plane charge transport.

**Table 3:** Summary of representative literature reports on additive-assisted crystallization and morphology modulation in functional organic thin films.

major component	minor additive	additive concentration	morphological mechanism	performance Impact	ref
**TES-ADT**	functionalized derivatives: **F-TES-ADT**, **diF-TES-ADT**, **Cl-TES-ADT**	0.5–2.5 mol %	heterogeneous seeding lowers nucleation barrier, yielding uniform spherulites	higher nuclei density; uniform grain size	^37^
	structurally distinct molecules: **TES-Pen**, **TIPS-Ant**, **Ethyl-TES-ADT**	>2.7 mol %	nonideal mixing disrupts host ordering, causing irregular crystallization	degrades morphology and control	
**TES-ADT**	**FTES-ADT**	0.6–6.3 mol %	increases nucleation density, shifting system to nucleation-dominated growth	mobility increases (5 × 10^−3^ to 3.6 × 10^−1^ cm^2^ V^−1^ s^−1^); grains shrink	^26^
**poly(*a*-methylstyrene** **)** or **PTAA)**	**TIPS-pentacene** or **diF-TESADT**	1:1 weight ratio	vertical phase separation enriches small molecules at the top interface	mobility significantly improved (1 × 10^−1^ to 2.4 cm^2^ V^−1^ s^−1^)	^27^
**PMMA**	**P3HT**	1–8 wt %	surface energy drives stratification, forming a P3HT-top/PMMA-bottom bilayer	optimal 5 wt % blend improves mobility	^28^
**P3HT**	**DAE-Me**	20 wt %	additives segregate into amorphous regions, expelled from ordered domains	affects transport vs switching trade-off	^11^
**BTBT** or **P3HT**	**DAE_Me** or **DAE_*t*Bu**	fixed at 20 wt % DAE (open form)	additives enrich at grain boundaries, creating tunable energy barriers	creates trap-controlled transport	^20^
**DPPT-TT**	**DAE_*t*Bu** or **DAE_F** or **DAE_*t*Bu + DAE_F**	10 wt % (or mix)	orbital-selective trapping targets specific carriers with minimal structural disruption	enables selective hole/electron modulation	^13^

Under meniscus-guided coating, the coexistence of pressure-driven and boundary-driven shear flow, capillary backflow, and Marangoni flow generates pronounced velocity, concentration, and temperature gradients near the moving meniscus.^29,41^ These coupled nonequilibrium fields align **PDVT-10** chains and preaggregated fibrils along the coating direction, while solvent evaporation progressively increases the local concentration at the solidification front. Within this dynamic zone, **DTCP** is proposed to remain predominantly dispersed and function as a morphology-modulating additive rather than as a seed-like heterogeneous nucleant. This distinction is substantiated by time-dependent UV–vis absorption measurements (Figures S16 and S17), which reveal no discernible spectral shifts or intensity changes in **DTCP** solutions over a 2 h period. The absence of temporal evolution in the absorption features confirms that **DTCP** does not undergo spontaneous self-aggregation or macroscopic phase separation in the precursor solution, thereby ruling out a classical precipitation-driven nucleation mechanism. Specifically, **DTCP** is expected to regulate local packing frustration and interfacial energetics at evolving growth interfaces, thereby promoting cooperative ordering and domain coalescence, consistent with the laterally connected stripe-like textures observed by AFM and EDX measurements. Importantly, the gradients inherent to MGC naturally imply spatially nonuniform ordering across the coated film. In agreement with this expectation, spatially resolved polarized UV measurements reveal modest but reproducible variations in dichroic response across different film regions, indicating region-dependent molecular orientation that reflects locally distinct solidification histories. Collectively, these results support an additive-regulated cooperative ordering pathway in which **DTCP** amplifies structural correlation in the precursor state and biases the gradient-driven assembly during MGC.^42–49^ However, because early stage nucleation in mixed small-molecule/polymer blends is inherently difficult to resolve, this study emphasizes robust structure–property correlations rather than assigning a definitive nucleation mechanism. Consequently, the proposed framework should be regarded as a working model and will require further multimodal validation to unambiguously disentangle the contributions of hydrodynamic alignment, evaporation-induced concentration gradients, and additive-mediated packing regulation.

### Electrical Characterization of DTCP-Doped PDVT-10 OFETs

To investigate the photoisomer-dependent electrical characteristics and molecular alignment, bottom-gate, top-contact OFETs were fabricated from MGC **PDVT-10** thin films blended with varying amounts of **DTCP-o** or **DTCP-c**. Source and drain electrodes were patterned on top of the films to define channels either parallel (∥) or perpendicular (⊥) to the coating direction (Figure 9). The results are summarized in Figure 10, Tables 4 and 5. Figure 10a shows the transfer characteristics of **PDVT-10**/**DTCP-o** devices. Upon introduction of **DTCP-o**, the drain current and hole mobility increase, with the 3 wt % blend exhibiting the highest mobility of 3.23 cm^2^ V^−1^ s^−1^ in the perpendicular direction, corresponding to ∼50% enhancement compared to pristine **PDVT-10** (2.12 cm^2^ V^−1^ s^−1^). Although **DTCP-o** is optically an insulating species with a large bandgap (4.26 eV), the energy diagram in Figure 10c reveals that its HOMO level (−5.77 eV) is relatively close to that of **PDVT-10** (−5.27 eV). This favorable HOMO alignment suggests that **DTCP-o** does not behave as a deep hole trap but can electronically communicate with **PDVT-10**, allowing interfacial hole transfer while primarily acting as a morphological regulator. At an optimal loading, **DTCP-o** assists MGC-induced directional coating by promoting backbone ordering, enlarging and aligning crystalline domains, and thereby improving charge transport. Consistently, **PDVT-10**/**DTCP-o** OFETs display an average *I*_on_/*I*_off_ ratio of approximately 10^4^, and the threshold voltage shifts from a relatively positive value (∼9 V) for the pure **PDVT-10** device toward ∼0 V after **DTCP-o** incorporation, which is advantageous for low-power operation. In contrast, Figure 10b shows that increasing the **DTCP-c** content leads to a gradual rise in drain current and mobility, reaching a maximum of 2.44 cm^2^ V^−1^ s^−1^ at 10 wt %, but this improvement is accompanied by pronounced hysteresis. The enlarged forward/reverse sweep offset at higher **DTCP-c** loadings indicates stronger charge trapping and interfacial disorder. The increased hysteresis in **DTCP-c** blends is consistent with the model proposed in previous literature report, where the closed-form isomer acts as a switchable charge trap due to its elevated HOMO level relative to the semiconductor matrix.^50^

**Figure 9:**
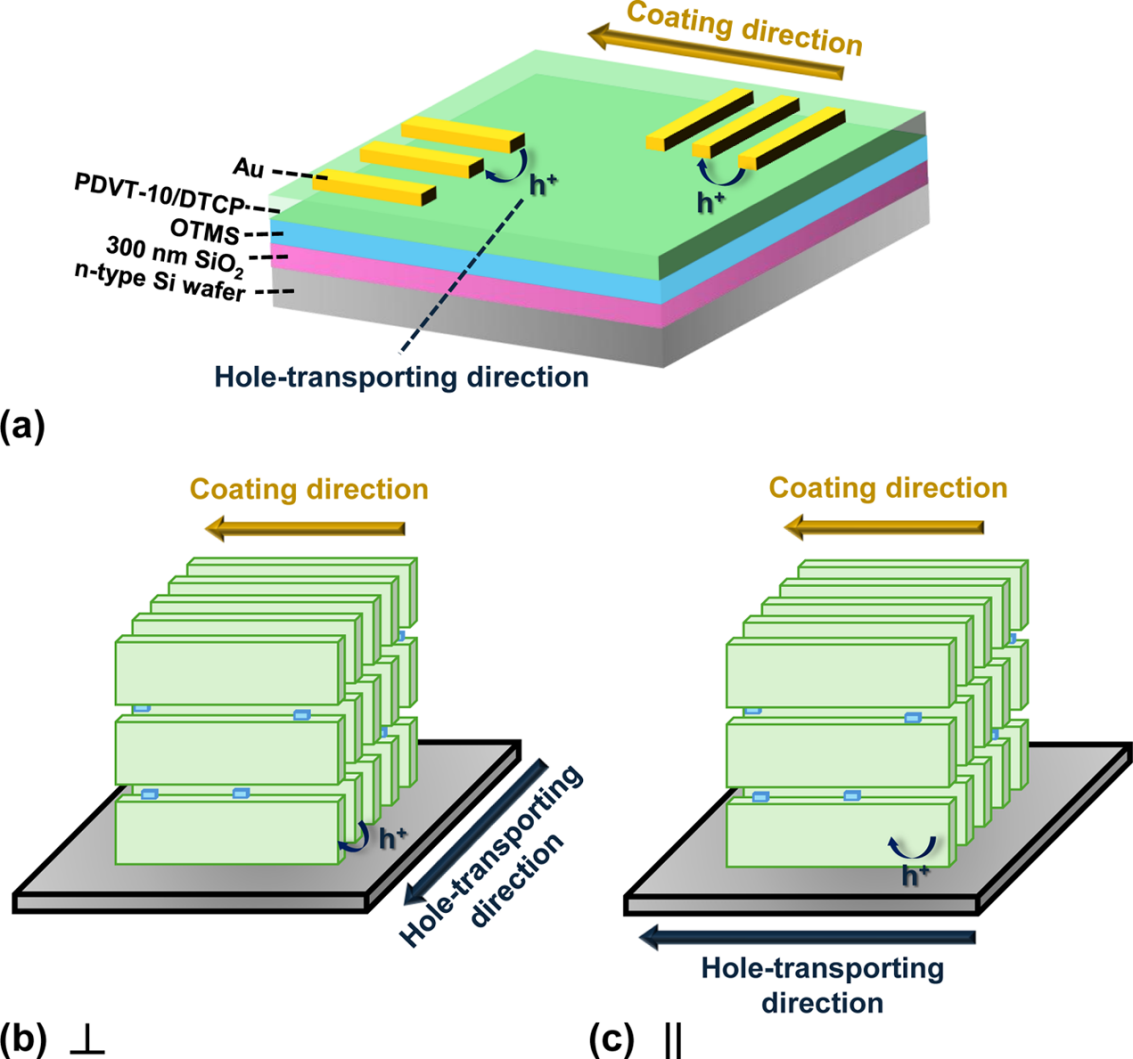
(a) Schematic of the MGC of **PDVT-10**/**DTCP** OFETs showing the coating direction and hole-transporting direction. Illustration of molecular alignment when hole transport occurs (b) perpendicular (⊥) and (c) parallel (||) to the coating direction.

**Figure 10:**
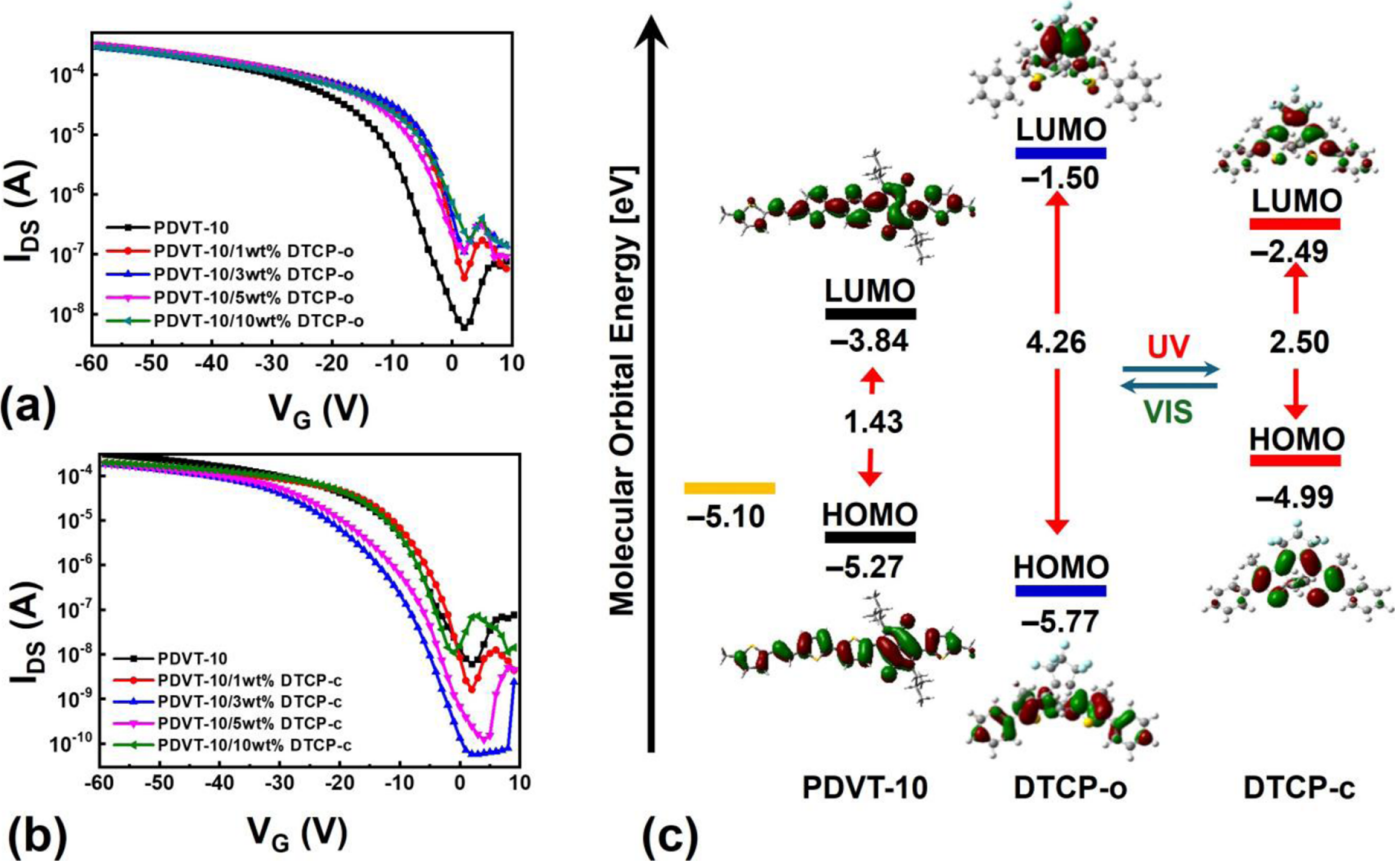
Transfer characteristics of OFET devices based on **PDVT-10** blended with various concentrations of (a) **DTCP-o** and (b) **DTCP-c**. (c) HOMO and LUMO energy levels of **PDVT-10**, **DTCP-o**, and **DTCP-c** calculated by DFT.

**Table 4:** Summary of the **PDVT-10** and **PDVT-10/DTCP-o** OFET Devices

coating direction	materials	mobility^max^ (cm^2^ V^−1^ s^−1^)	mobility^avg^ (cm^2^ V^−1^ s^−1^)	*I*_on_/*I*_off_	V_th_^avg^ (V)
⊥	**PDVT-10**	2.12	1.67 ± 0.21	9.57 × 10^3^	9.06 ± 1.61
	**PDVT-10/1 wt % DTCP-o**	2.86	2.00 ± 0.61	1.28 × 10^4^	−0.17 ± 0.54
	**PDVT-10/3 wt % DTCP-o**	3.23	2.59 ± 0.21	3.07 × 10^3^	1.11 ± 0.30
	**PDVT-10/5** **wt % DTCP-o**	2.17	1.88 ± 0.17	4.57 × 10^3^	−1.30 ± 0.76
	**PDVT-10/10 wt % DTCP-o**	2.12	1.40 ± 0.35	2.33 × 10^3^	1.99 ± 0.63
||	**PDVT-10**	0.73	0.39 ± 0.24	9.68 × 10^3^	7.11 ± 0.96
	**PDVT-10/1** **wt % DTCP-o**	1.17	0.90 ± 0.15	1.40 × 10^4^	−1.23 ± 0.63
	**PDVT-10/3 wt % DTCP-o**	1.68	1.20 ± 0.18	1.69 × 10^3^	4.26 ± 0.34
	**PDVT-10/5** **wt % DTCP-o**	1.21	0.81 ± 0.21	6.86 × 10^3^	−0.61 ± 0.97
	**PDVT-10/10** **wt % DTCP-o**	1.16	0.89 ± 0.05	4.72 × 10^3^	0.55 ± 2.54

**Table 5:** Summary of the **PDVT-10** and **PDVT-10/DTCP-c** OFET Devices

coating direction	materials	mobility^max^ (cm^2^ V^−1^ s^−1^)	mobility^avg^ (cm^2^ V^−1^ s^−1^)	*I*_on_/*I*_off_	V_th_^avg^ (V)
⊥	**PDVT-10**	2.12	1.67 ± 0.21	9.57 × 10^3^	9.06 ± 1.61
	**PDVT-10/1** **wt % DTCP-c**	2.25	1.85 ± 0.24	4.31 × 10^5^	−5.06 ± 0.56
	**PDVT-10/3 wt % DTCP-c**	1.70	1.28 ± 0.24	1.27 × 10^6^	−11.3 ± 2.08
	**PDVT-10/5** **wt % DTCP-c**	1.75	1.44 ± 0.17	5.36 × 10^5^	−10.04 ± 1.53
	**PDVT-10/10** **wt % DTCP-c**	2.44	1.94 ± 0.24	1.62 × 10^4^	−5.18 ± 0.60
||	**PDVT-10**	0.73	0.39 ± 0.24	9.68 × 10^3^	7.11 ± 0.96
	**PDVT-10/1 wt % DTCP-c**	1.24	0.87 ± 0.26	5.69 × 10^4^	−5.43 ± 2.52
	**PDVT-10/3 wt % DTCP-c**	1.13	0.92 ± 0.13	1.93 × 10^6^	−7.28 ± 0.77
	**PDVT-10/5** **wt % DTCP-c**	0.99	0.89 ± 0.05	2.12 × 10^4^	−9.12 ± 1.26
	**PDVT-10/10** **wt % DTCP-c**	1.52	1.19 ± 0.34	1.48 × 10^5^	−4.96 ± 1.10

### Photoswitching Behavior of PDVT-10/DTCP Hybrid OFETs

Figure 11 and Table S3 summarize the photoswitching behavior of the OFETs under alternating visible and UV irradiation. A closer examination of the mobility evolution during individual UV and visible-light irradiation steps reveals that the mobility change associated with each UV irradiation step can be quantified by the parameter Δ_*i*_^UV^ = UV_*i*_/VIS_*i*−1_ − 1, which directly compares the mobility after UV irradiation with that immediately preceding UV exposure (Table S4). For the neat **PDVT-10** device, the normalized hole mobility gradually decreases with repeated light exposure, indicating a monotonic photoinduced degradation of charge transport (Figure 11a). This UV irradiation step parameter remains relatively small and irregular across cycles for neat **PDVT-10** (Δ_*i*_^UV^ **=** −5.90, −13.37, −7.07, −7.96, −7.32% for cycles 1 to 5, respectively), suggesting that UV irradiation induces only a weak and nonsystematic mobility change that is largely masked by the overall photodegradation of the polymer backbone. In contrast, the Δ_*i*_^UV^ of the transistor based on **PDVT-10/3 wt % DTCP** hybrid (Figure 11b) exhibits significantly larger negative values (**−**13.60, −26.47, −13.51, −14.62, −13.74% for cycles 1 to 5, respectively), demonstrating a reproducible and cycle-independent mobility decrease induced by UV irradiation that is consistently and stronger than that observed in neat **PDVT-10**. This behavior indicates that the UV irradiation step in the hybrid device introduces an additional photoresponsive process beyond intrinsic polymer degradation, which can be attributed to the UV-induced structural transformation of the **DTCP** component.

**Figure 11:**
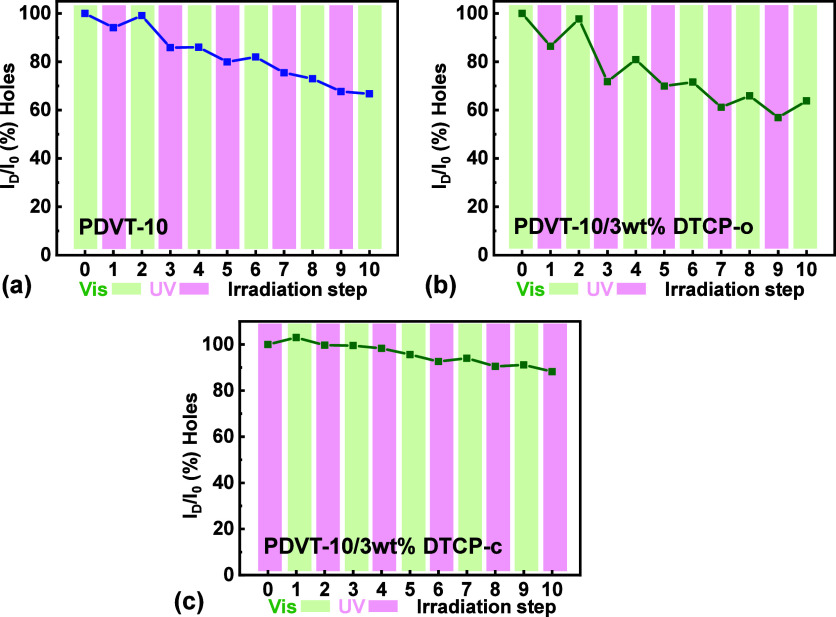
Photoswitching behavior of **PDVT-10** and **PDVT-10**/**3 wt % DTCP** hybrid OFETs under alternating visible and UV irradiation. (a) Normalized hole mobility of the neat **PDVT-10** device as a function of switching cycle number. (b) **PDVT-10/3 wt % DTCP-o** hybrid device measured with an initial visible-light step (starting from the stable open-ring state). (c) **PDVT-10/3 wt % DTCP-c** hybrid device measured with an initial UV-light step (starting the cycle by converting to the closed-ring state). The green and pink shaded regions indicate periods of Vis and UV exposure, respectively.

Meanwhile, the mobility change associated with the visible-light irradiation step is described by the parameter Δ_*i*_^VIS^ = VIS_*i*_/UV_*i*−1_ − 1, which compares the mobility after visible-light irradiation with that immediately following UV exposure within the same cycle. For pure **PDVT-10**, Δ_*i*_^VIS^ rapidly diminishes with increasing cycle number and becomes negative in later cycles (Δ_*i*_^VIS^ = 5.32, 0.16, 2.55, −3.25, and −1.33%), indicating that visible-light irradiation is no longer able to counteract the UV-induced mobility loss once cumulative photodegradation dominates the device response. By contrast, the **PDVT-10/3 wt % DTCP** exhibits consistently positive Δ_*i*_^VIS^ values (13.14, 12.56, 2.43, 7.78, and 12.18% for cycles 1 to 5, respectively), although noticeable fluctuations are observed across cycles. These fluctuations can be attributed to the interplay between the reversible photoisomerization kinetics of **DTCP** and slower processes such as trap formation, charge detrapping, or local microstructural relaxation within the hybrid film. Importantly, the persistence of positive Δ_*i*_^VIS^ values demonstrates that visible-light irradiation remains effective in partially restoring charge transport in the hybrid device, even as the absolute mobility gradually decays with repeated cycling.

To further evaluate the photoswitching behavior at the level of a complete irradiation cycle, the cycle-by-cycle modulation depth *D*_*i*_ = (VIS_*i*_-UV_*i*_)/VIS_*i*_ is introduced to quantify the net mobility modulation retained after each UV–visible irradiation cycle (Table S5). For the neat **PDVT-10** device, *D*_*i*_ rapidly decreases with increasing cycle number and approaches zero within the first few cycles (*D*_*i*_ **=** 5.05, 0.16, 2.49, −3.36, −1.35% for cycles 1 to 5, respectively). This rapid loss of cycle-level modulation indicates that, although small stepwise mobility changes may still be detected, the device no longer maintains a meaningful net electrical tunability after a complete irradiation cycle. In this case, the apparent photoresponse is dominated by cumulative photochemical or structural degradation of the polymer, rather than a reversible switching process.

In contrast, the **PDVT-10/3 wt % DTCP** exhibits a substantially larger and more persistent cycle modulation depth over repeated irradiation cycles (*D*_*i*_ **=** 11.61, 11.16, 2.37, 7.22, 10.86% for cycles 1 to 5, respectively). Although a temporary reduction of *D*_*i*_ is observed in intermediate cycles, the modulation depth partially recovers in subsequent cycles and remains significantly higher than that of neat **PDVT-10** throughout the entire cycling process. This behavior reflects the combined effect of the UV and visible-light irradiation steps: while gradual baseline mobility decay is unavoidable, the reversible photochromic transformation of **DTCP** preserves a substantial fraction of the cycle-level electrical tunability. Notably, the modulation of charge carrier mobility arises from the synergistic interaction between the electronic trap formation discussed earlier and the morphological changes induced by in situ isomerization. The transition between the twisted open-ring and planar closed-ring isomers involves significant alterations in molecular geometry and free volume requirements.^51^ Within a densely packed solid-state film, such conformational shifts unavoidably generate local steric stress, which can disrupt the coherent π–π stacking of the host polymer matrix. In the optimized hybrid system (3 wt % **DTCP**), however, the bulky and flexible alkyl side chains of **PDVT-10** are hypothesized to function as a steric buffer, accommodating these geometric variances and preserving the integrity of the crystalline domains. While this buffering capacity facilitates the observed reversible switching, the cumulative accumulation of microstructural disorder resulting from repetitive lattice expansion and contraction cannot be entirely excluded. This morphological fatigue, acting in concert with the intrinsic photochemical degradation of the polymer, likely contributes to the gradual attenuation of the on/off modulation amplitude observed over extended cycling. As a result, *D*_*i*_ provides a significant quantitative indicator that distinguishes a photosensitive but nonswitchable transistor into one that can be actively toggled between distinct conductivity states by light, highlighting the promise of **DTCP**-based hybrids as photoresponsive, optically switchable organic electronic platforms for future device applications. To elucidate the origin of the gradual mobility decay observed during repeated photoswitching cycles, we evaluated the photostability of the individual components. Diarylethene derivatives are well-established for their exceptional fatigue resistance, with reported systems sustaining over 10,000 switching cycles with minimal performance loss.^52,53^ This suggests that intrinsic photochemical exhaustion of the **DTCP** dopant is unlikely to be the primary cause of the degradation. Regarding the polymer host, while the original reports on **PDVT-10** demonstrated its high charge carrier mobility, they did not explicitly investigate its stability under the intense, alternating UV/visible irradiation conditions required for photoswitching.^21,54,55^ Our control experiments on neat **PDVT-10** devices (Figure 11a) fill this gap, revealing a measurable monotonic decrease in mobility and on-current upon repeated illumination even in the absence of the dopant. These results indicate that the **PDVT-10** polymer matrix or the semiconductor/dielectric interface is susceptible to photoinduced degradation, which constitutes the limiting factor for the long-term cycling endurance of the hybrid devices.

To further validate the reliability of the photoswitching mechanism, we performed a complementary reverse-sequence measurement (Figure 11c), where the irradiation cycle was initiated with UV exposure (converting the initial open-ring state to the closed-ring state). Consistent with the standard visible-first sequence, the hybrid device exhibited distinct and reversible on/off switching, confirming that the optical modulation is intrinsic to the **DTCP** molecules and independent of the irradiation history. Interestingly, the device exposed to the UV-first sequence displayed a notably lower baseline degradation rate compared to the standard visible-first measurement. This enhanced stability suggests that the specific cycling protocol influences device fatigue. Initiating with trap formation (UV step) followed immediately by detrapping/recovery (visible step) may mitigate the accumulation of irreversible deep traps or morphological stress typically associated with extended dwell times in the initial measurement states. The preservation of switching magnitude in both sequences reinforces the robustness of the **PDVT-10/DTCP** hybrid system for versatile optoelectronic operations.

## Conclusions

In this work, blending the conjugated polymer **PDVT-10** with the photochromic small molecule **DTCP** is shown to be an effective approach for tuning film microstructure and OFET characteristics in meniscus-guided coated devices. By systematically varying **DTCP** loading in both the open (**DTCP-o**) and closed (**DTCP-c**) forms, it is found that the two isomers influence shear- and evaporation-coupled film formation in distinct ways. At an optimized **DTCP-o** content, the **PDVT-10/DTCP-o** hybrid films deliver improved field-effect mobility, reduced threshold voltage, and minimal hysteresis. These improvements are attributed to a favorable energetic landscape, including HOMO level proximity between **PDVT-10** (−5.27 eV) and **DTCP-o** (−5.77 eV), together with **DTCP-o** acting primarily as a morphology-regulating additive that enhances backbone ordering and crystalline coherence under MGC, consistent with the aggregation and ordering trends identified by the structural and solution-phase analyses. In contrast, incorporation of the more planar **DTCP-c** can yield a higher peak mobility, but it also introduces pronounced hysteresis and sweep-direction-dependent threshold shifts at higher loadings. These behaviors are consistent with reduced miscibility and stronger aggregation propensity of **DTCP-c**, leading to **DTCP-c**-enriched features that can strengthen local electronic coupling while simultaneously increasing trapping and interfacial disorder. Integrating the electrical, structural, and energetic results, a **DTCP**-regulated cooperative crystallization framework is proposed, in which pressure-driven shear, capillary backflow, and Marangoni flow near the moving meniscus align **PDVT-10** fibrils along the coating direction. Meanwhile, **DTCP** modulates packing and interfacial organization during solidification, rather than acting as a seed-like nucleant. Overall, this study highlights photochromic small molecules as versatile additives for regulating morphology and transport in shear-coated conjugated polymer semiconductors, providing practical guidance for designing scalable, high-performance organic electronic devices based on controlled nonequilibrium assembly.

## Supplementary Material



## Notes

Contents comprise: (i) energy-level calculations of **PDVT-10**, **DTCP-o**, and **DTCP-c**; (ii) OM/POM images of **PDVT-10** and **DTCP**-blended films prepared under various coating speeds, substrate gaps, and concentrations; (iii) 2D-GIXRD patterns and coherence-length calculations for lamellar and π–π stacking; (iv) DLS analyses; (v) AFM surface roughness analyses; (vi) polarized UV–Vis absorption spectra; and (vii) complete OFET transfer/output curves and mobility extraction (PDF)
