# Supplementary material for: Morphology Control in PDVT-10/DTCP Hybrid Films via Meniscus-Guided Cooperative Crystallization for High-Performance OFETs
Source: ACS Appl Mater Interfaces. 2026 Feb 4;18(6):10338–53. doi: 10.1021/acsami.5c23619 (PMC12926940; doi:10.1021/acsami.5c23619)
Supplement: Supplementary file 1 [file am5c23619_si_001.pdf]

Supporting Information

Morphology Control in PDVT-10/DTCP Hybrid Films via Meniscus-Guided Cooperative Crystallization for High-Performance OFETs

Xiao-Yuan Lin,[a] Dhananjay S. Nipate,[a] Shih-Kang Chen,[a] Mai Harada,[b] U-Ser Jeng,[c] Michal Kohout,[d] Hong-Cheu Lin,[e,f] Yasutaka Kitagawa,[b] Tomoyuki Akutagawa,[g,h,*] Wen-Ya Lee,[i,*] Hsiu-Hui Chen[a,*]

[a]Department of Molecular Science and Engineering, National Taipei University of Technology, Taipei 106, Taiwan

[b]Department of Materials Engineering Science, Graduate School of Engineering Science, Osaka University, Osaka, 560-8531, Japan

[c]National Synchrotron Radiation Research Center, Hsinchu City 300, Taiwan

[d]Department of Organic Chemistry, University of Chemistry and Technology Prague, Prague 16628, Czech Republic

[e]Department of Materials Science and Engineering, National Yang Ming Chiao Tung University, Hsinchu 300093, Taiwan

[f]Center for Emergent Functional Matter Science, National Yang Ming Chiao Tung University, Hsinchu 300093, Taiwan

[g]Graduate School of Engineering, Tohoku University, Sendai 980-8579, Japan

[h]Institute of Multidisciplinary Research for Advanced Materials (IMRAM), Tohoku University, 2-1-1 Katahira, Aoba-ku, Sendai 980-8577, Japan

[i]Department of Chemical Engineering and Biotechnology, National Taipei University of Technology, Taipei 106, Taiwan

Corresponding Author

Hsiu-Hui Chen: hhchen@mail.ntut.edu.tw

Wen-Ya Lee: wenyalee@mail.ntut.edu.tw

Tomoyuki Akutagawa: tomoyuki.akutagawa.b5@tohoku.ac.jp

Table of Contents

**Contents**	**Page**
Film thickness and energy-level calculations (**Tables S1–S2**)	3
OM and POM analysis of PDVT-10/DTCP films (**Figures S1–S5**)	4
Molecular orientation and 2D-GIXRD analysis (**Figures S6–S7**)	9
Crystallite coherence length and size distributions (**Figures S8–S10**)	11
Solution-state DLS analysis (**Figures S11**)	13
AFM surface morphology and roughness analysis (**Figure S12**)	14
Polarized UV–Vis absorption and optical anisotropy (**Figures S13–S14**)	15
Solution-state UV-Vis and aggregation behavior (**Figures S15–S17**)	17
OFET output, transfer curves, and photoswitching data (**Figures S18–S19**; **Tables S3–S5**)	19

Table S1. Average thickness of the **PDVT-10** and **PDVT-10/DTCP** hybrid films.

Samples	Average thickness (nm)
**PDVT-10**	112.45 ± 1.03
**PDVT-10/1 wt % DTCP-o**	96.67 ± 3.41
**PDVT-10/3 wt % DTCP-o**	115.00 ± 0.73
**PDVT-10/5 wt % DTCP-o**	114.26 ± 1.47
**PDVT-10/10 wt % DTCP-o**	100.57 ± 0.70
**PDVT-10/1 wt % DTCP-c**	114.88 ± 2.16
**PDVT-10/3 wt % DTCP-c**	115.91 ± 0.43
**PDVT-10/5 wt % DTCP-c**	109.99 ± 1.79
**PDVT-10/10 wt % DTCP-c**	117.29 ± 0.77

Average thickness of all films: 110.78 ± 7.09

Table S2. Summary of the energy levels of HOMO and LUMO, as well as the energy gaps, for **PDVT-10**, **DTCP-o**, and **DTCP-c**.

	**PDVT-10**	**DTCP-o**	**DTCP-c**
	B3LYP/6-31G*	B3LYP/6-31G*	B3LYP/6-31G*
LUMO [eV]	–2.574	–1.503	–2.494
HOMO [eV]	–4.639	–5.765	–4.993
H-L gap [eV]	2.06	4.26	2.50

2.1 OM and POM of PDVT-10 and DTCP-Blended Films

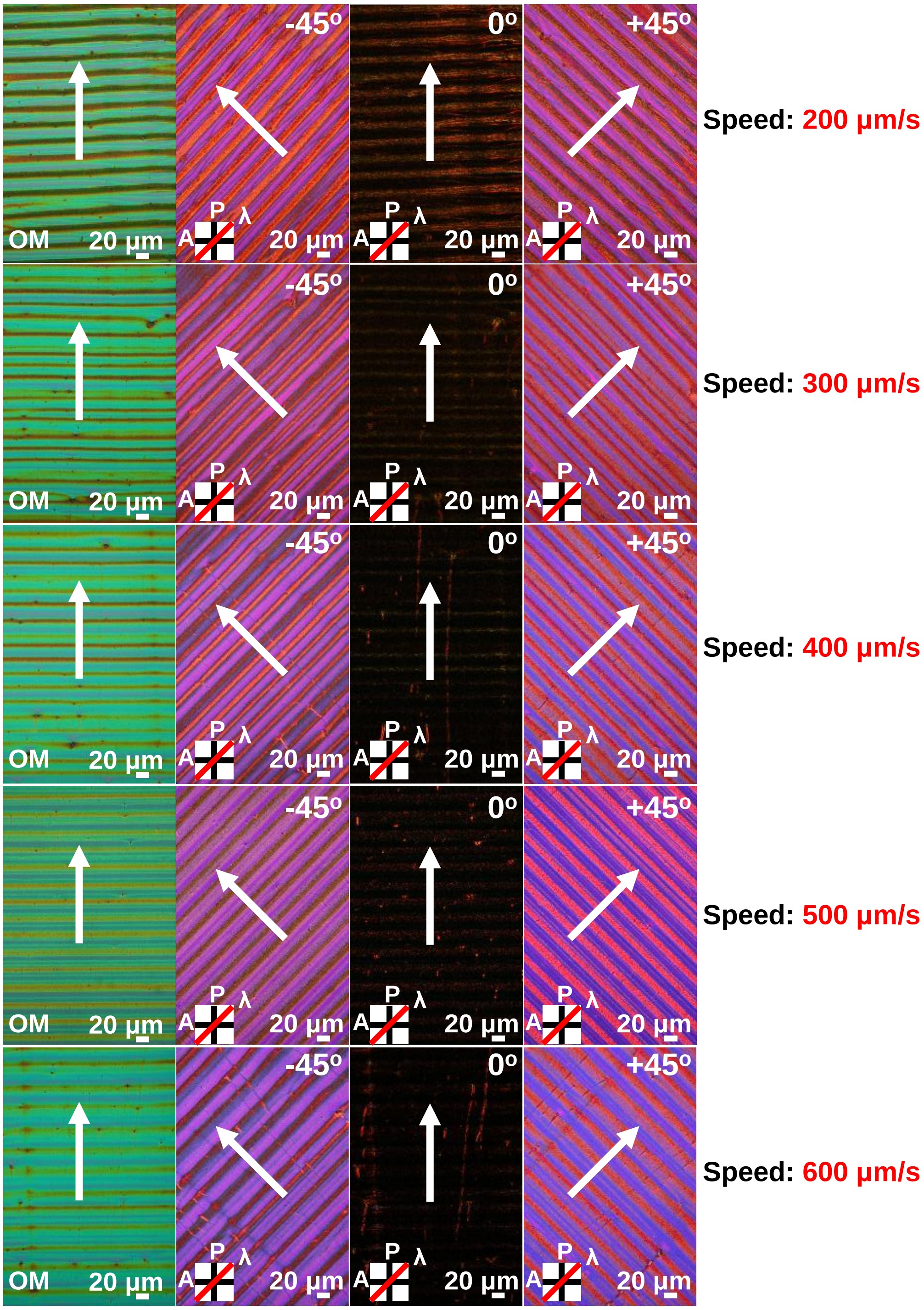

Figure S1. Optical microscopy (OM) and polarized optical microscopy (POM) images of **PDVT-10** thin films were prepared using different coating speeds of 200, 300, 400, 500, and 600 μm/s under identical coating conditions. The white arrows in the images indicate the coating direction.

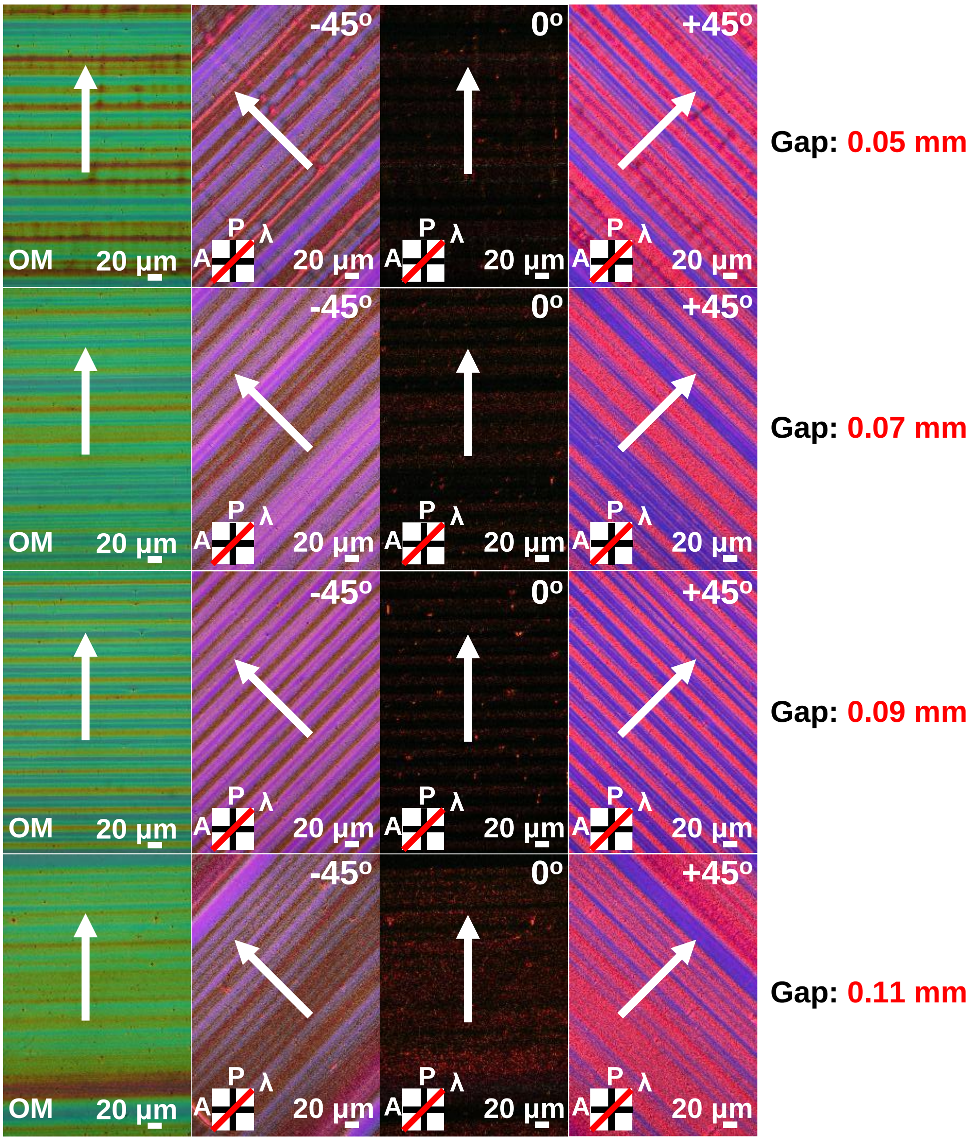

Figure S2. OM and POM images of **PDVT-10** thin films prepared with different blade-to-substrate gaps of 0.05, 0.07, 0.09, and 0.11 mm under identical coating conditions. White arrows in the images are the coating direction.

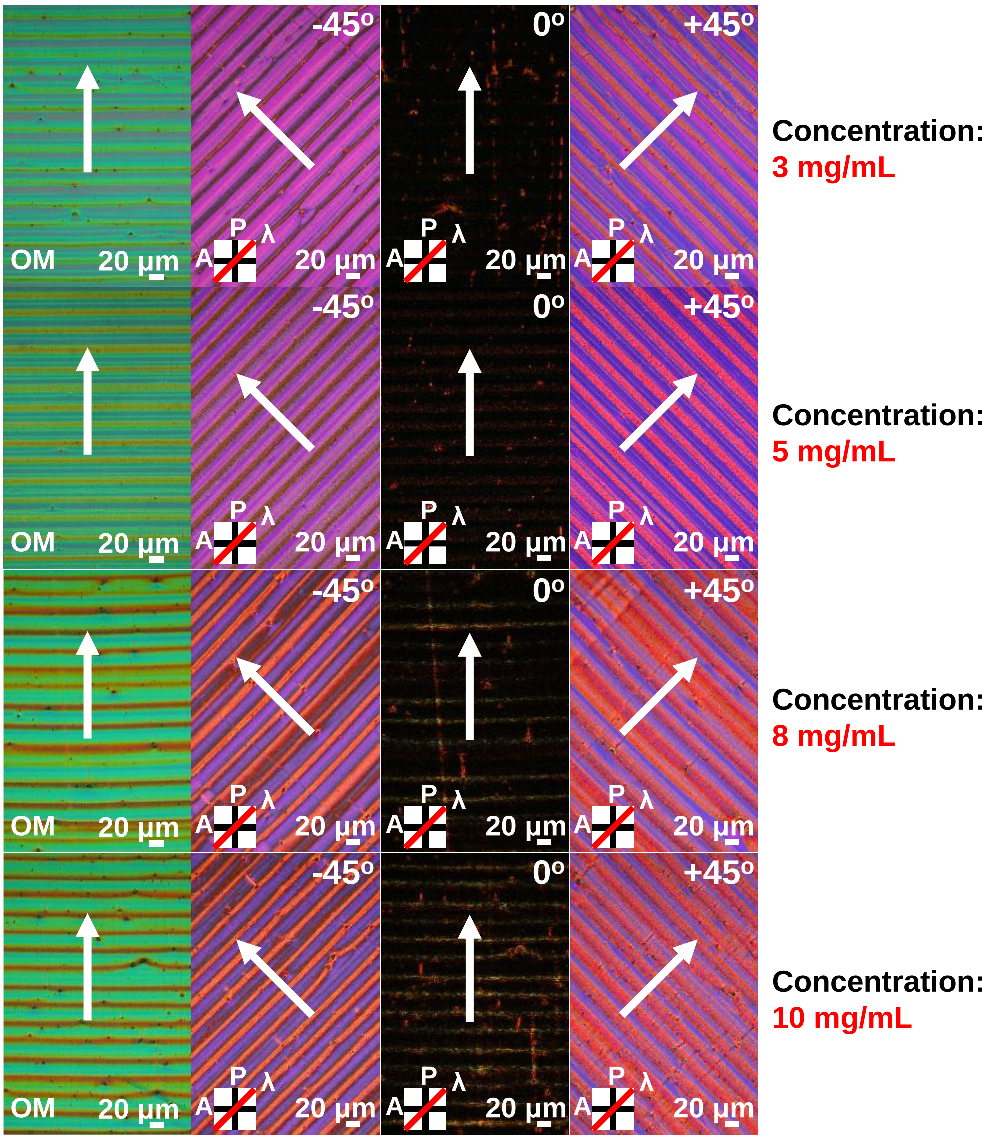

Figure S3. OM and POM images of **PDVT-10** thin films prepared with different concentrations of 3, 5, 8, and 10 mg/mL under identical coating conditions. White arrows in the images are the coating direction.

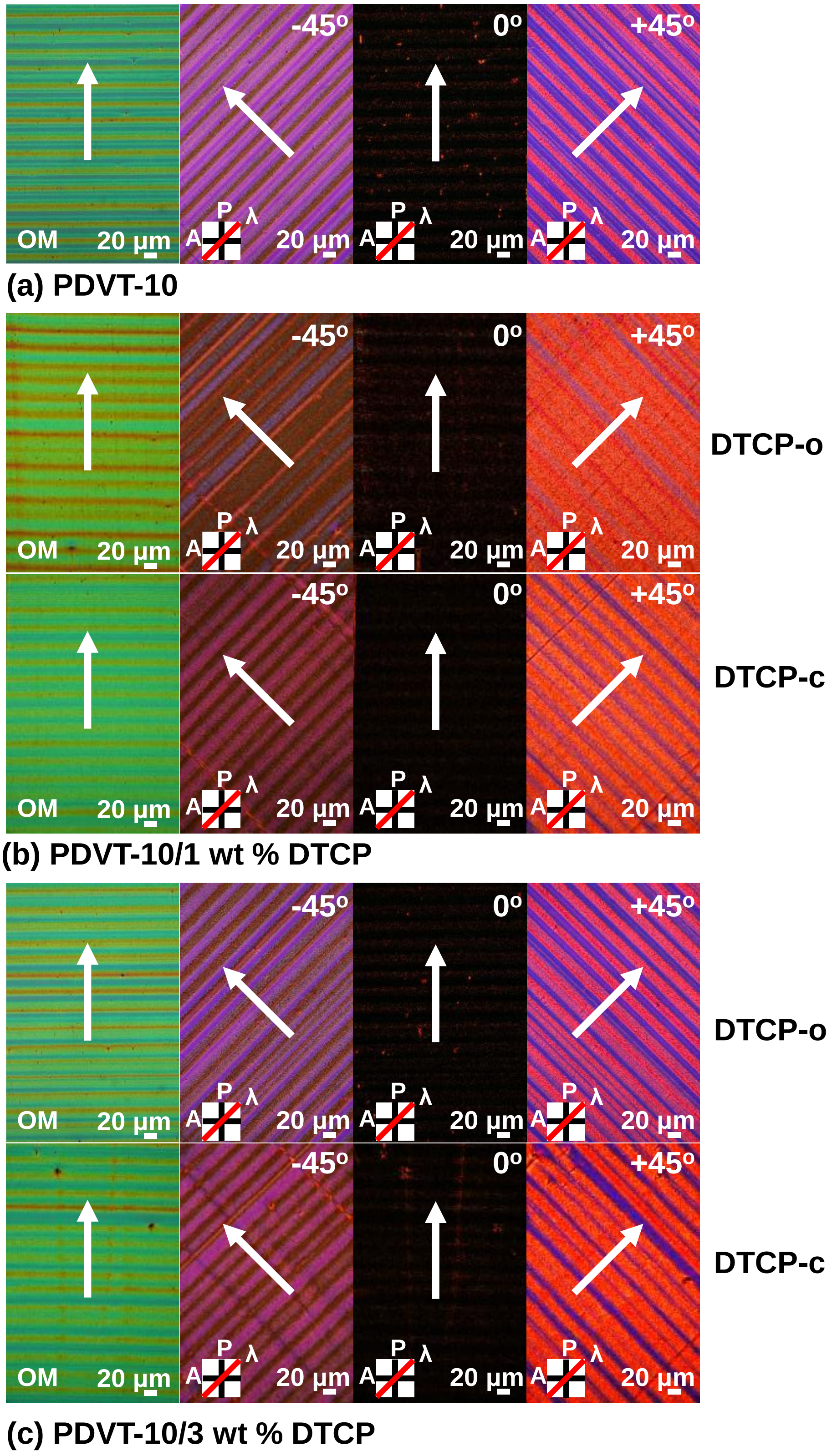

Figure S4. OM and POM images for coated thin films of **PDVT-10** blends with (a) 0 wt %, (b) 1 wt %, and (c) 3 wt % **DTCP-o/ DTCP-c** under different polarization angles (0°, ± 45°) with a λ = 530 nm waveplate. White arrows in the images are the coating direction.

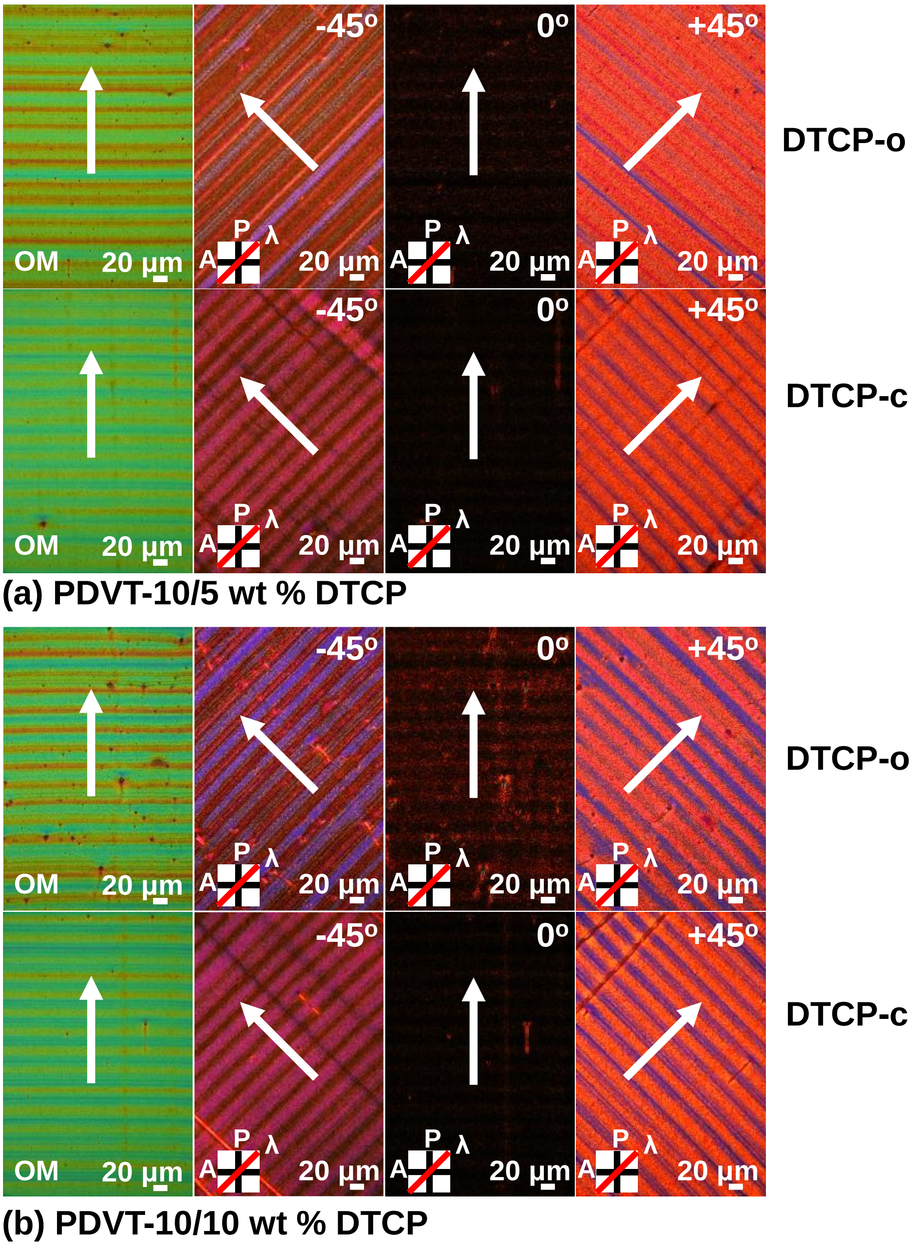

Figure S5. OM and POM images for coated thin films of **PDVT-10** blends with (a) 5 wt % and (b) 10 wt % **DTCP-o/ DTCP-c** under different polarization angles (0°, ± 45°) with a λ = 530 nm waveplate. White arrows in the images are the coating direction.

2.2 Molecular Orientation and Crystallographic Alignment

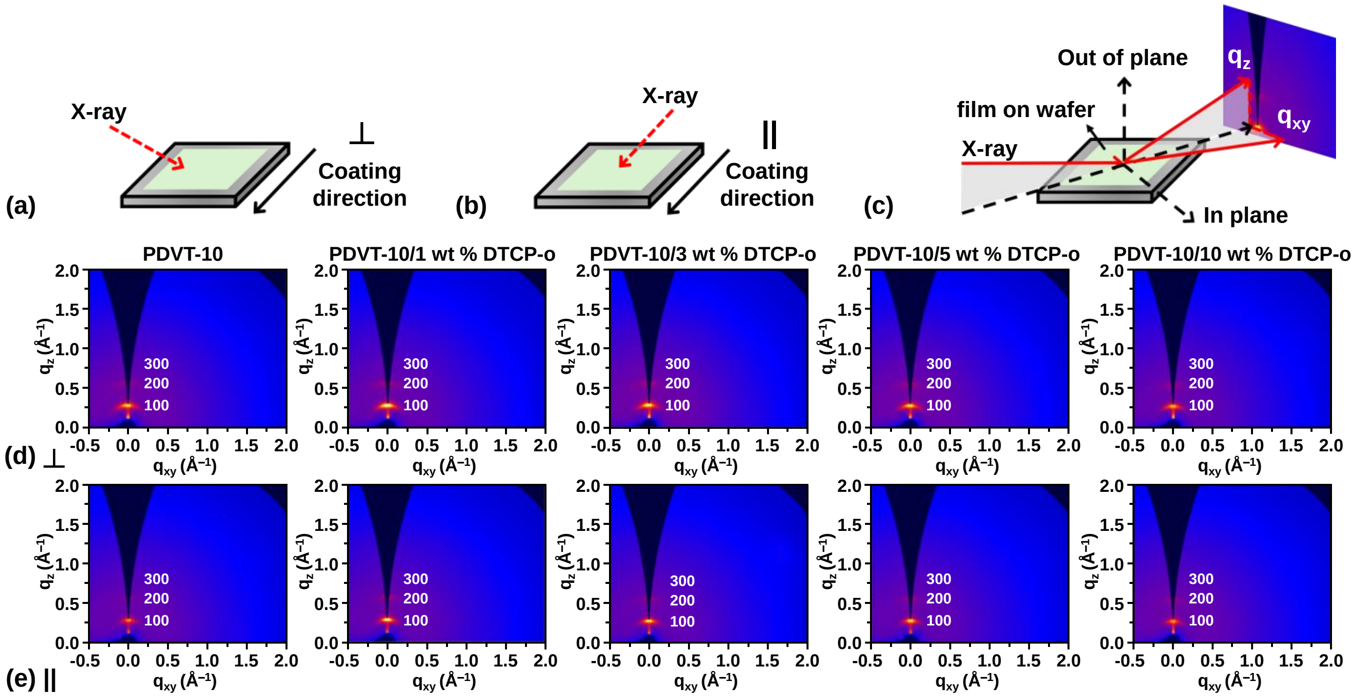

Figure S6. Illustration of (a) perpendicular (⊥) and (b) parallel (||) orientation of the X-ray incidence beam with respect to the coating direction. (c) Schematic of the 2D-GIXRD measurement on PDVT/DTCP samples at an incidence angle of 0.12°. 2D-GIXRD patterns from (d) perpendicular and (e) parallel measurements on the following samples: **PDVT-10**, **PDVT-10/1 wt % DTCP-o**, **PDVT-10/3 wt % DTCP-o, PDVT-10/5 wt % DTCP-o**, and **PDVT-10/10 wt % DTCP-o**.

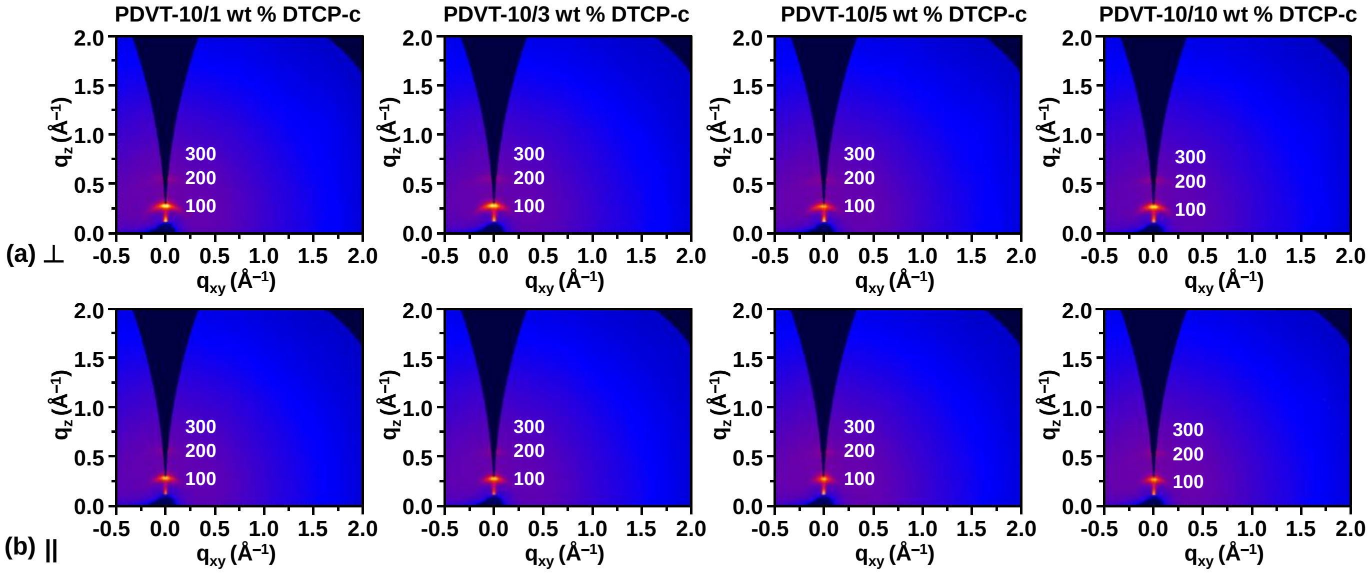

Figure S7. 2D-GIXRD patterns from (a) perpendicular and (b) parallel measurements on the following samples: **PDVT-10**, **PDVT-10/1 wt % DTCP-c**, **PDVT-10/3 wt % DTCP-c, PDVT-10/5 wt % DTCP-c**, and **PDVT-10/10 wt % DTCP-c**. (The perpendicular (⊥) and parallel (||) refer to the relative orientation of the X-ray incidence beam with respect to the coating direction.)

2.3 Calculation of coherence length along the (100), (200), and (010) direction. The coherence length was calculated by the Scherrer equation, as shown in eq 1:

$$\mathrm{L} = K\lambda/\mathrm{W}_{1/2}\cos\theta \qquad (1)$$

where L is the size of the crystallite in Å, K is a dimensionless shape factor approximately 0.89, λ is the incident X-ray wavelength of 1.02745 Å, $\mathrm{W}_{1/2}$ is the full width at half maximum (FWHM) of the diffraction peak in radians, θ is the Bragg angle in radians.

The drain current (I_{DS}) was calculated in the saturation regime (V_{DS} = −60 V) from the transfer curves, according to the following eq 2:

$$I_{\mathrm{DS}} = \mu_{\mathrm{FET}} W C_{\mathrm{g}}(\mathrm{V}_{\mathrm{G}}-\mathrm{V}_{\mathrm{th}})^2/2L \qquad (2)$$

where W, L, C_{g}, V_{G}, and V_{th} represent the channel width (2000 µm), channel length (100 µm), capacitance per unit area of the gate dielectric layer (10 nF cm^2), gate voltage, and threshold voltage, respectively. The field-effect mobility (μ_{FET}) is extracted using eq 3, as shown below:

$$\mu_{\mathrm{FET}} = (2I_{\mathrm{DS}}L)/(WC_{\mathrm{g}}(\mathrm{V}_{\mathrm{G}}-\mathrm{V}_{\mathrm{th}})^2) \qquad (3)$$

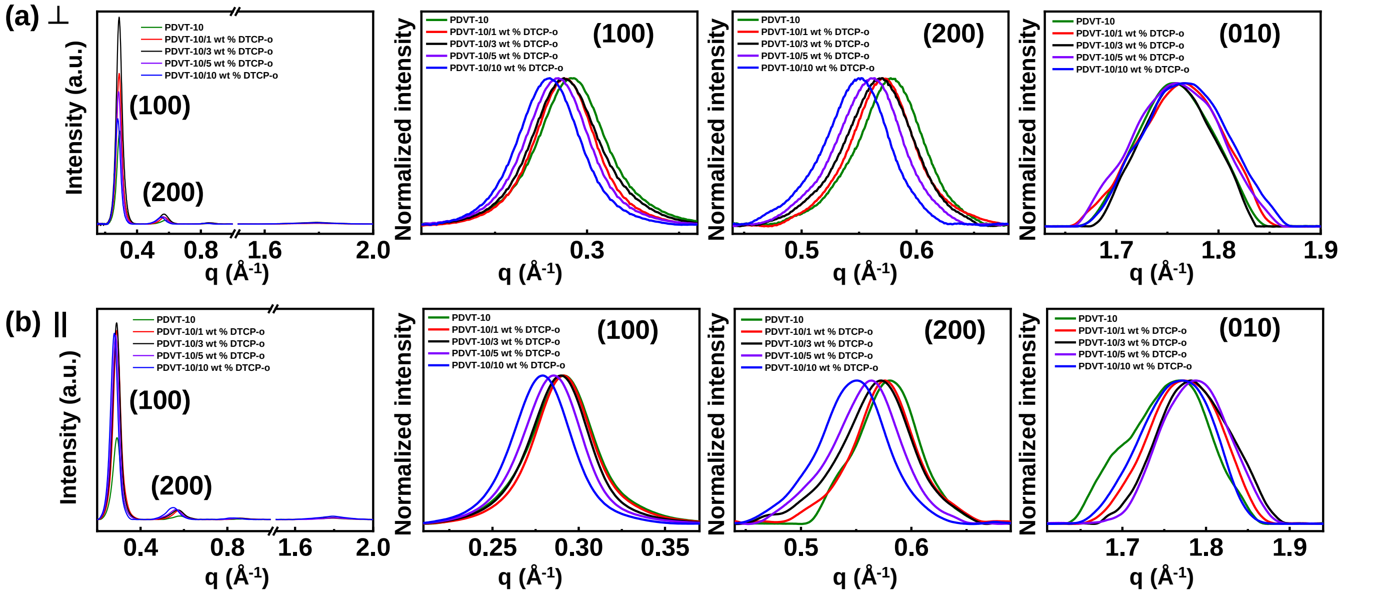

Figure S8. GIXRD patterns of **PDVT-10/DTCP-o** thin films with (a) perpendicular (⊥) and (b) parallel (||) X-ray beam orientations relative to the coating direction, showing (100) and (200) lamellar stacking peaks as well as in-plane π–π stacking peaks.

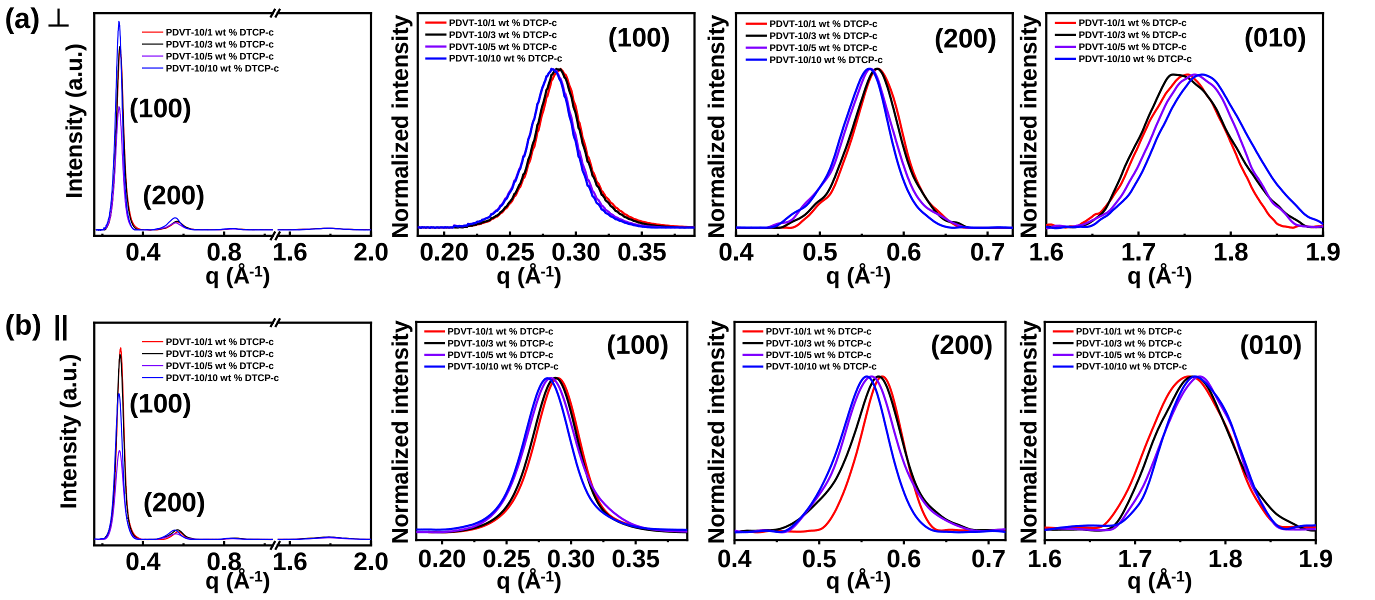

Figure S9. GIXRD patterns of **PDVT-10/DTCP-c** thin films with (a) perpendicular (⊥) and (b) parallel (||) X-ray beam orientations relative to the coating direction, showing (100) and (200) lamellar stacking peaks as well as in-plane π–π stacking peaks.

2.4 Volume-weighted crystallite size distributions

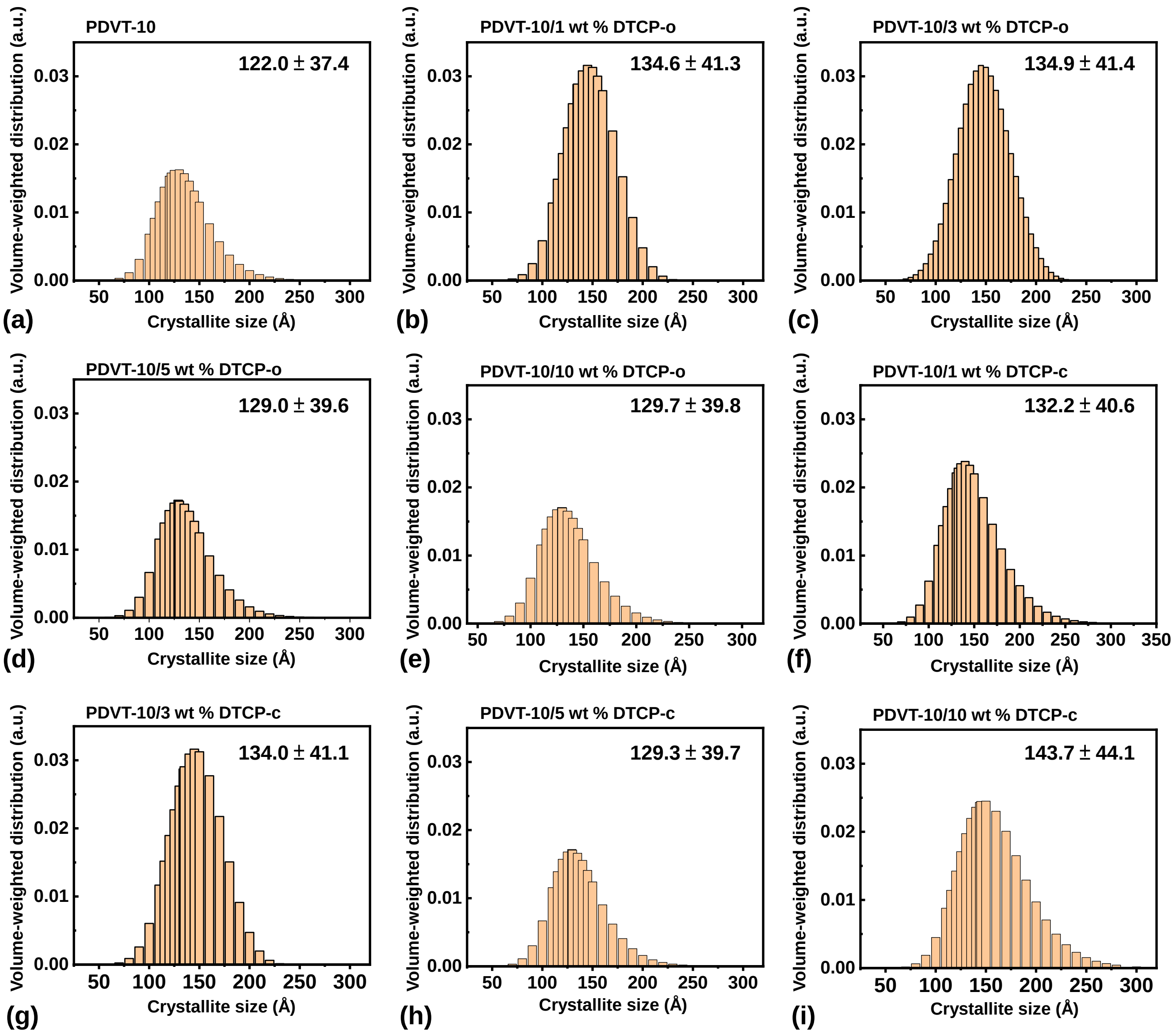

Figure S10. Volume-weighted crystallite size distributions reconstructed from GIXRD data for (a) **PDVT-10**, (b) **PDVT-10/1wt% DTCP-o**, (c) **PDVT-10/3 wt % DTCP-o**,(d) **PDVT-10/5 wt % DTCP-o**, (e) **PDVT-10/10 wt % DTCP-o**, (f) **PDVT-10/1 wt % DTCP-c**, (g) **PDVT-10/3 wt % DTCP-c**, (h) **PDVT-10/5 wt % DTCP-c**, and (i) **PDVT-10/10 wt % DTCP-c**, derived from Scherrer coherence lengths with a polydispersity index ($\sigma = 0.3$).

2.5 Solution-state DLS analysis

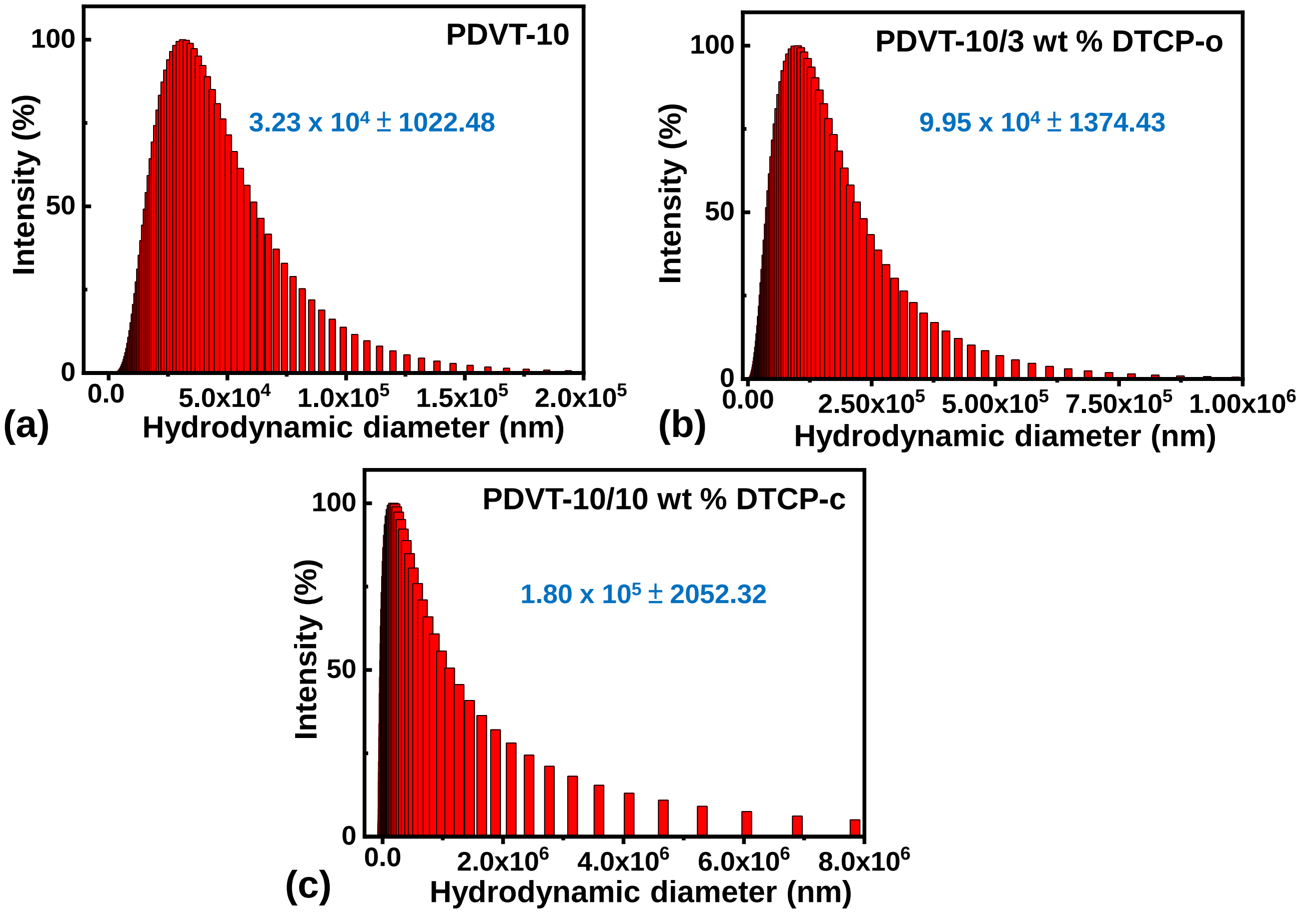

Figure S11. DLS intensity-weighted size distributions of (a) neat **PDVT-10**, (b) **PDVT-10/3 wt % DTCP-o**, and (c) **PDVT-10/10 wt % DTCP-c** solutions. All measurements were conducted in chloroform at a dilute concentration of 2.75×10^{-7} M.

2.6 Surface Morphology

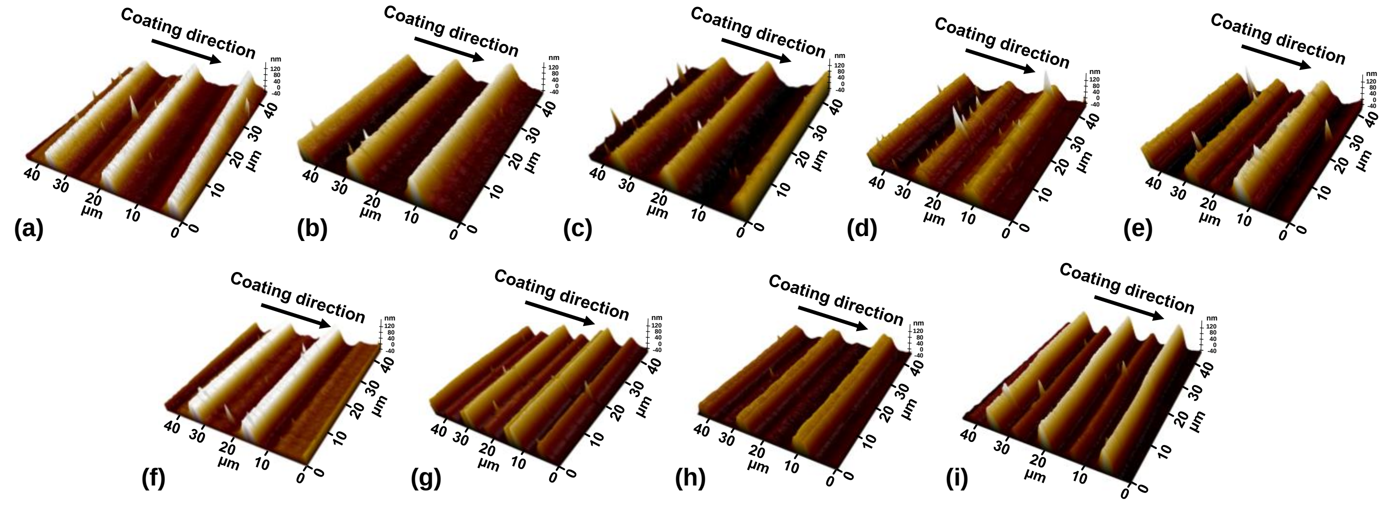

Figure S12. The AFM micrograph of (a) **PDVT-10**, (b) **PDVT-10/1 wt % DTCP-o**, (c) **PDVT-10/3 wt % DTCP-o**, (d) **PDVT-10/5 wt % DTCP-o**, (e) **PDVT-10/10 wt % DTCP-o**, (f) **PDVT-10/1 wt % DTCP-c**, (g) **PDVT-10/3 wt % DTCP-c**, (h) **PDVT-10/5 wt % DTCP-c, and** (i) **PDVT-10/10 wt % DTCP-c** films.

The arithmetic average roughness (R_a) is determined using eq 4, as shown below:

$$Ra = \frac{1}{N}\sum_{i=1}^{N}|z_i-\bar{z}| \qquad (4)$$

where z_i is the height value of the i-th pixel, $\bar{z}$ is the average height of the entire image (or the selected analysis region), and N is the total number of pixels included in the calculation.

The root-mean-square roughness (R_q) is determined using eq 5, as shown below:

$$R_q \approx 1.1{\sim}1.3 \times R_a \qquad (5)$$

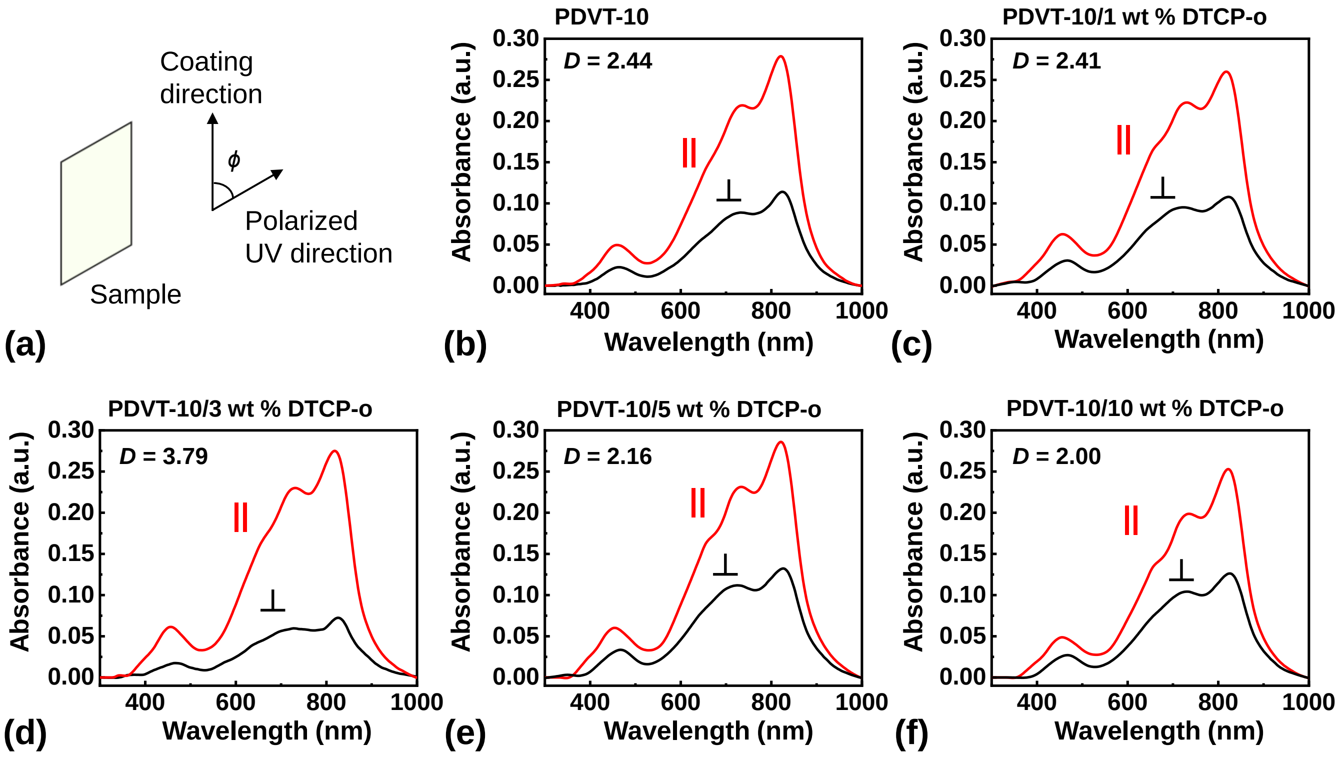

Figure S13. (a) Illustration of the coating direction and the measurement geometry for polarized UV light in the sample. Polarized UV–Vis absorption spectra of (b) **PDVT-10**, (c) **PDVT-10/1 wt % DTCP-o**, (d) **PDVT-10/3 wt % DTCP-o**, (e) **PDVT-10/5 wt % DTCP-o**, and (f) **PDVT-10/10 wt % DTCP-o** films. The red and black curves correspond to absorption measured with the light polarization parallel (||) and perpendicular (⊥) to the coating direction, respectively.

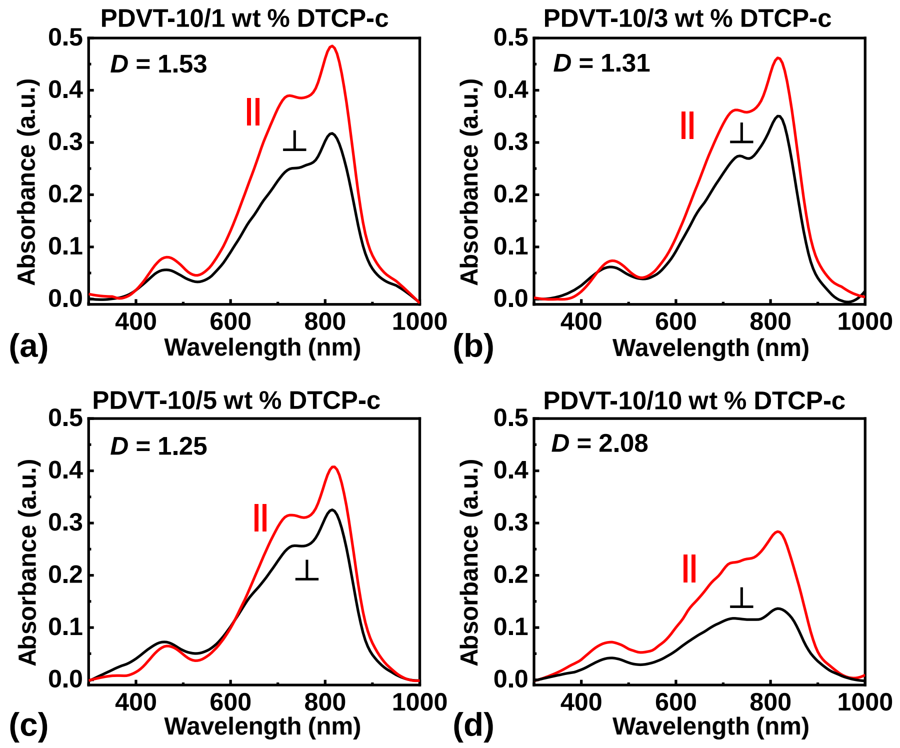

Figure S14. Polarized UV–Vis absorption spectra of (a) **PDVT-10/1 wt % DTCP-c**, (b) **PDVT-10/3 wt % DTCP-c**, (c) **PDVT-10/5 wt % DTCP-c**, and (d) **PDVT-10/10 wt % DTCP-c** films. The red and black curves correspond to absorption measured with the light polarization parallel (||) and perpendicular (⊥) to the coating direction, respectively.

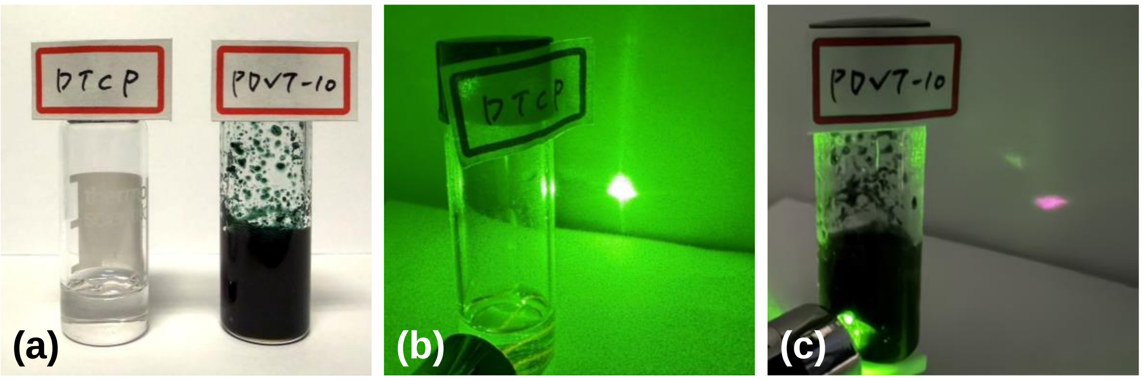

Figure S15. Visual observation of the solution states. (a) Photographs of the **DTCP** (left) and **PDVT-10** (right) solutions. Images of (b) the **DTCP** solution and (c) the **PDVT-10** solution upon laser irradiation.

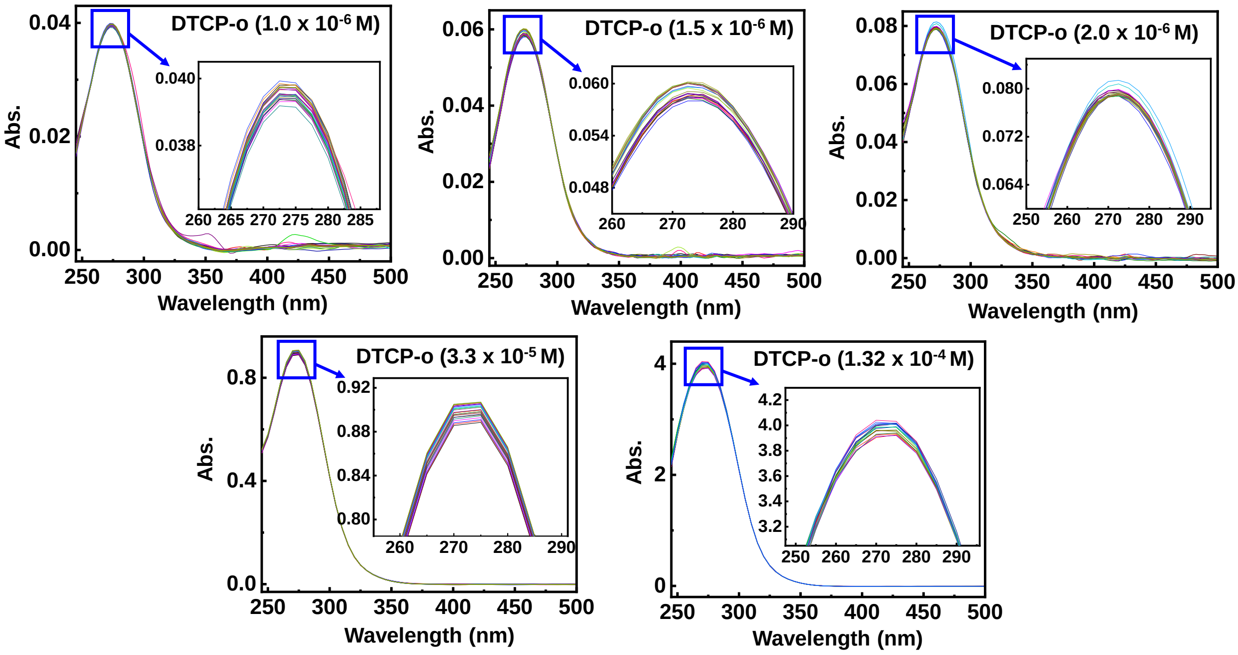

Figure S16. Time-dependent UV–Vis absorption spectra of **DTCP** solutions at different concentrations, recorded under static conditions at 5 m intervals for up to 2 h.

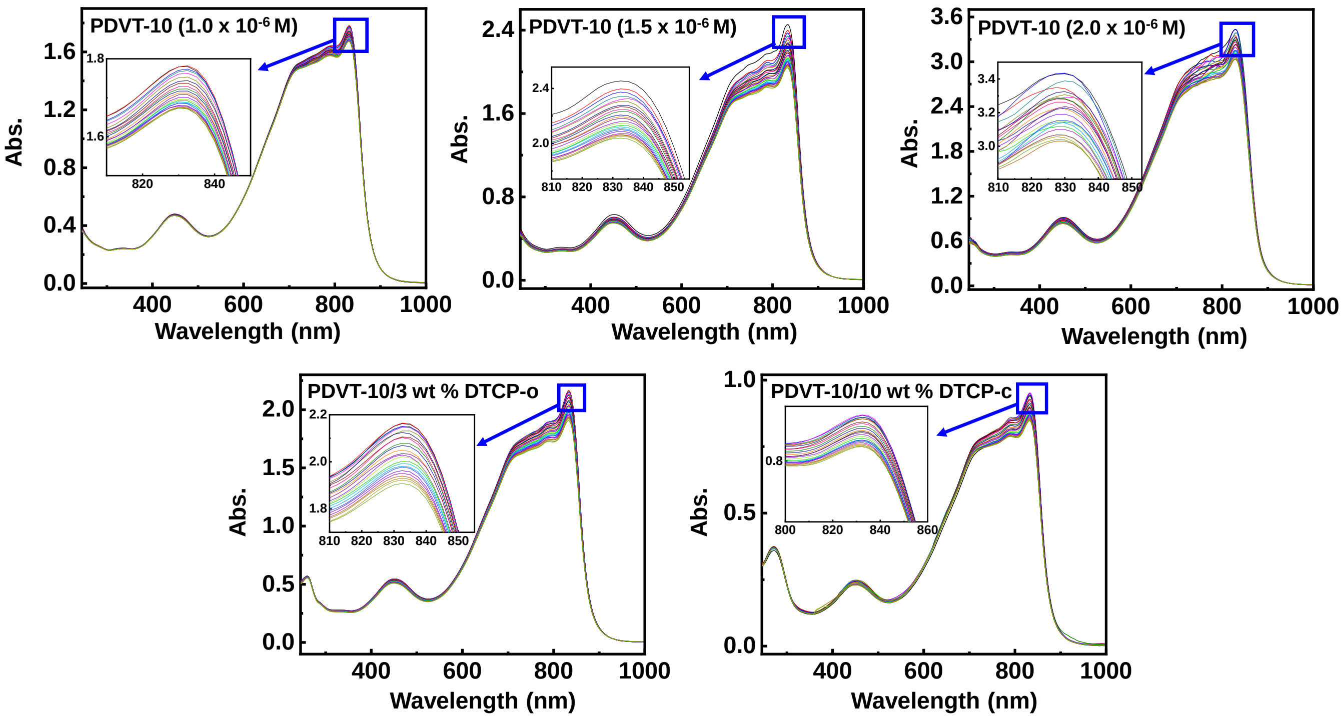

Figure S17. Time-dependent UV–Vis absorption spectra of **PDVT-10**, **PDVT-10/DTCP** solutions at different concentrations, recorded under static conditions at 5 min intervals for up to 2 h.

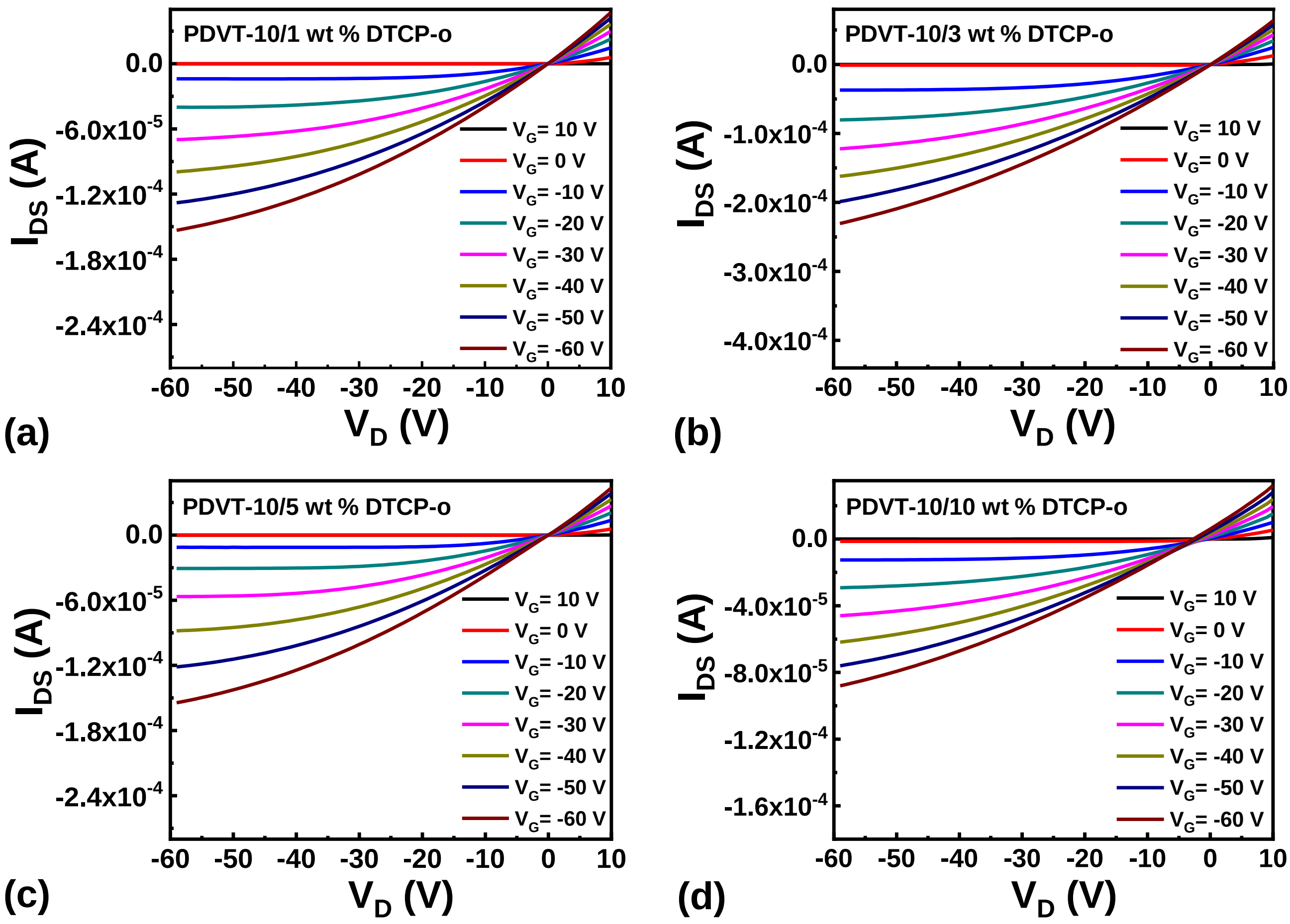

Figure S18. The output curves of (a) **PDVT-10/1 wt % DTCP-o**, (b) **PDVT-10/3 wt % DTCP-o**, (c) **PDVT-10/5 wt % DTCP-o**, and (d) **PDVT-10/10 wt % DTCP-o**.

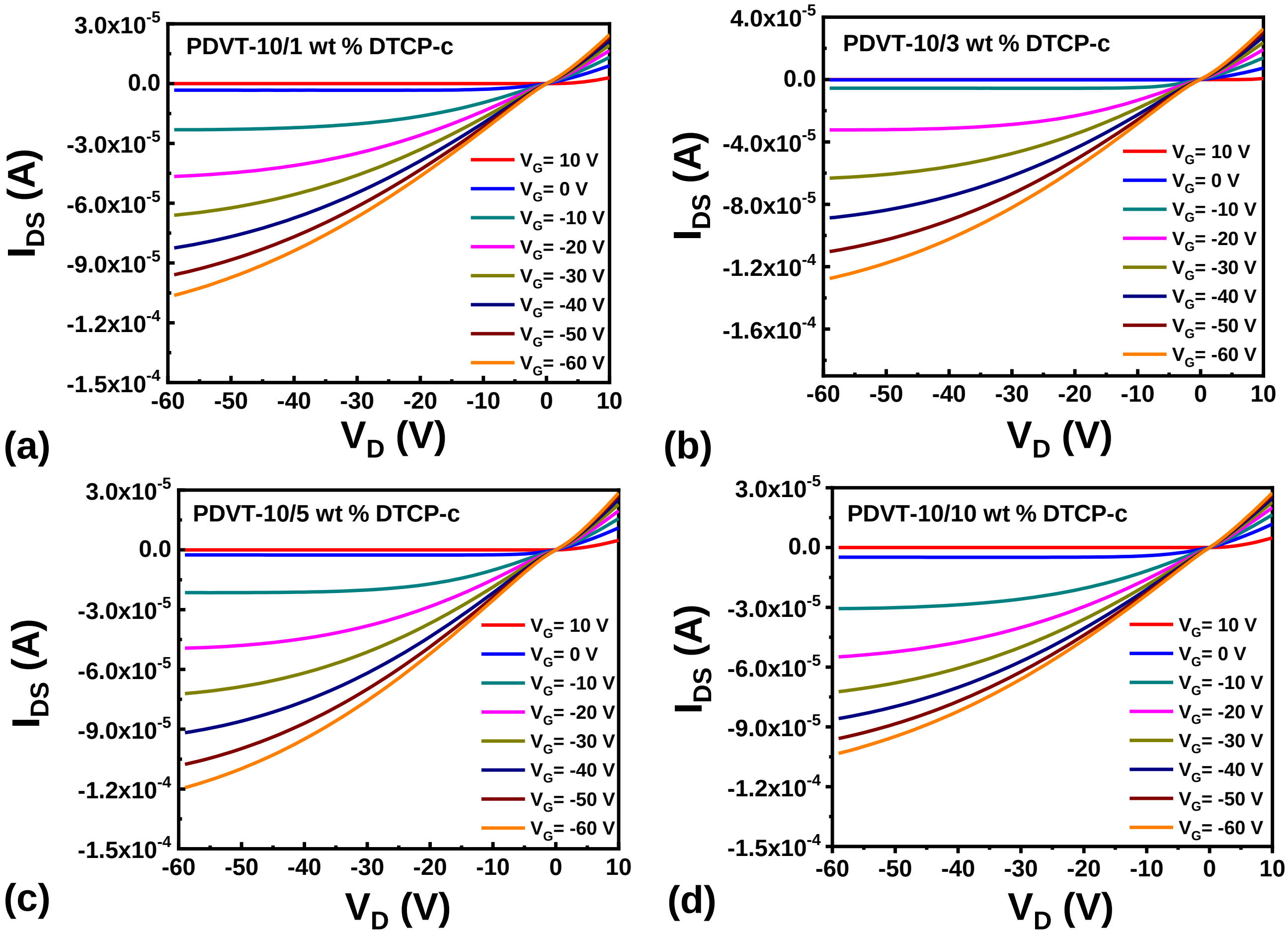

Figure S19. The output curves of (a) **PDVT-10/1 wt % DTCP-c**, (b) **PDVT-10/3 wt % DTCP-c**, (c) **PDVT-10/5 wt % DTCP-c**, and (d) **PDVT-10/10 wt % DTCP-c**.

Table S3. Normalized drain current (I_D/I_0, %) of **PDVT-10** and **PDVT-10/3 wt % DTCP** OFET devices as a function of irradiation step under alternating visible (Vis) and ultraviolet (UV) light exposure. The values are extracted from the photoswitching experiments, where I_D/I_0 is normalized to the initial current measured before irradiation (step 0).

Irradiation step	I_D/I_0 (%)	
	**PDVT-10**	**PDVT-10/3 wt % DTCP**
0	100.00	100.00
1	94.10	86.40
2	99.11	97.75
3	85.86	71.88
4	86.00	80.91
5	79.92	69.98
6	81.96	71.68
7	75.44	61.20
8	72.99	65.96
9	67.65	56.90
10	66.75	63.83

Table S4. Step–by–step mobility changes of **PDVT-10** and **PDVT-10/3 wt % DTCP** OFETs during individual UV and visible-light irradiation steps under alternating irradiation.

Cycle	UV irradiation step (%)		Vis irradiation step (%)	
	**PDVT-10**	**PDVT-10/3 wt % DTCP**	**PDVT-10**	**PDVT-10/3 wt % DTCP**
1	–5.90	–13.60	5.32	13.14
2	–13.37	–26.47	0.16	12.56
3	–7.07	–13.51	2.55	2.43
4	–7.96	–14.62	−3.25	7.78
5	–7.32	–13.74	−1.33	12.18

Table S5. Cycle–by–cycle net mobility modulation of **PDVT-10** and **PDVT-10/3 wt % DTCP** OFETs over successive UV–visible irradiation cycles under alternating irradiation.

Cycle	Modulation depth (%)	
	**PDVT-10**	**PDVT-10/3 wt % DTCP**
1	5.05	11.61
2	0.16	11.16
3	2.49	2.37
4	–3.36	7.22
5	–1.35	10.86
